# Marine Extremophiles: A Source of Hydrolases for Biotechnological Applications

**DOI:** 10.3390/md13041925

**Published:** 2015-04-03

**Authors:** Gabriel Zamith Leal Dalmaso, Davis Ferreira, Alane Beatriz Vermelho

**Affiliations:** 1 BIOINOVAR—Biotechnology laboratories: Biocatalysis, Bioproducts and Bioenergy, Institute of Microbiology Paulo de Góes, Federal University of Rio de Janeiro, Av. Carlos Chagas Filho, 373, 21941-902 Rio de Janeiro, Brazil; E-Mail: gdalmaso@gmail.com; 2 Graduate Program in Plant Biotechnology, Health and Science Centre, Federal University of Rio de Janeiro, Av. Carlos Chagas Filho, 373, 21941-902 Rio de Janeiro, Brazil; 3 BIOINOVAR—Biotechnology Laboratories: Virus-Cell Interaction, Institute of Microbiology Paulo de Góes, Federal University of Rio de Janeiro, Av. Carlos Chagas Filho, 373, 21941-902 Rio de Janeiro, Brazil; E-Mail: davisf@micro.ufrj.br

**Keywords:** enzymes, marine extremophiles, hydrolases

## Abstract

The marine environment covers almost three quarters of the planet and is where evolution took its first steps. Extremophile microorganisms are found in several extreme marine environments, such as hydrothermal vents, hot springs, salty lakes and deep-sea floors. The ability of these microorganisms to support extremes of temperature, salinity and pressure demonstrates their great potential for biotechnological processes. Hydrolases including amylases, cellulases, peptidases and lipases from hyperthermophiles, psychrophiles, halophiles and piezophiles have been investigated for these reasons. Extremozymes are adapted to work in harsh physical-chemical conditions and their use in various industrial applications such as the biofuel, pharmaceutical, fine chemicals and food industries has increased. The understanding of the specific factors that confer the ability to withstand extreme habitats on such enzymes has become a priority for their biotechnological use. The most studied marine extremophiles are prokaryotes and in this review, we present the most studied archaea and bacteria extremophiles and their hydrolases, and discuss their use for industrial applications.

## 1. Extremophiles from Marine Habitats: A Source of Bioproducts

The oceans cover more than 70% of the surface of the planet Earth and contain a vast biological diversity, accounting for more than 95% of the whole biosphere. Marine habitats can be divided into coastal and open ocean environments of various natures, supporting marine life with a wide heterogeneity of microorganisms. They represent the largest reservoir of biodiversity on the planet, and have a great potential for the development of new natural products including enzymes. However, in the prokaryotic group only 1%–10% of the species has been described [1]. 

Extreme environments combine a range of physical parameters such as pressure, temperature, pH, salinity, oxidative stress, radiation, chemicals (oxygen, H_2_S, CH_4_) and metals (Fe, Cu, Mo, Zn, Cd, Pb and others). Some of these chemicals are toxic, however extremophiles microorganisms are able to adapt due to their highly flexible metabolism, allowing them to survive and thrive in these extreme conditions [2]. Over the last few decades, extremophiles have attracted the attention of research centers in the search for new bioactive substances, such as enzymes and biocides to be used in major sectors of the world economy, including the agricultural, chemical, food, textile, pharmaceutical, bioenergy and cosmetic industries.

The global market for industrial enzymes was nearly US$ 4.8 billion in 2013, and it is expected to reach US$ 7.1 billion by 2018, with a compound annual growth rate (CAGR) of 8% over the five-year period, according to BCC Research [3]. Besides their economic value, microbial enzymes are applied in technologies employing eco-friendly processes [4]. Microorganisms are easily cultivated in bioreactors with controllable growth conditions such as pH, temperature, aeration, medium composition and other parameters, leading to high reproducibility. In contrast, enzymes isolated from plant and animals present a series of limitations like soil composition, light incidence, seed homogeneity, pathogen control and other issues that make the reproducibility of these processes more difficult [5].

Competition for space and nutrients in the marine environment constitutes a selective force leading to evolution and generating multiple enzyme systems to adapt to the different environments. Marine microorganisms can be found in extreme conditions such as hypersaline habitats, high pressures and extreme temperature. Many marine extremophiles are capable of overcoming such extreme conditions and are a source of enzymes with special characteristics. Therefore, these microorganisms are of great interest for industrial processes, mainly in biocatalysis [6]. This vast variation in marine habitats has led to the development of new hydrolases with novel specificities and properties including tolerance to extreme conditions used in industrial processes [7,8]. Metagenomic studies have revealed that extremophile prokaryotes from marine habitats are a source of novel genes and consequently a source of new bioproducts, including enzymes and other active metabolites. Therefore, it is important to study and understand these microorganisms in order to be able to use the biochemical, ecological, evolutionary and industrial potential of these marine microbes [9,10].

Hydrolases from extremophiles have advantages when compared to chemical biocatalysts. Their catalyses are clean processes, ecologically friendly, highly specific and take place in mild reaction conditions. These hydrolases can also be active in the presence of organic solvents, an important feature for the preparation of single-isomer chiral drugs. Various applications have been described for these hydrolases. A metagenome library was created from the brine: seawater interface of the Urania hypersaline basin. One esterase presented high enantioselectivity toward an ester of the important chiral synthon solketal, an important drug intermediate. The esterase hydrolyzed solketal acetate, producing building blocks for the synthesis of pharmaceuticals, such as anti-AIDS drugs [11]. Lipases from psychrophiles can be used for the synthesis of a wide range of nitrogenized compounds that are used for the production of pharmaceuticals such as amines and amides. This procedure was studied in *Candida Antarctica*, a patented eukaryote [12].

Other hydrolases with medical applications are peptidases. They are useful catalysts for inorganic synthesis and have many industrial applications in the pharmaceutical field, such as anti-inflammatory and digestive agents. Peptidases can be isolated from marine extremophiles [10], such as from *Thermatoga maritime*, which is a hyperthermophilic isolate from a marine geothermal area near Vulcano, Italy, and has a homomultimeric peptidase (669 kDa) based on 31 kDa subunits, named Maritimacin [13]. This enzyme was found to have a structural and gene sequence similarity to bacteriocin from the mesophilic bacterium *Brevibacterium linens* which inhibits the growth of certain Gram-positive bacteria [14]. The thermophilic *Pyrococcus horikoshii* has an intracellular peptidase (PH1704) with remarkable stability. Recently this enzyme, a cysteine peptidase, was shown to be the first allosteric enzyme that has negative cooperativity with chloride ions (Cl^−^). The discovery of new allosteric sites is very important for pharmaceutical developments [15]. Peptidases from halophiles have been used in peptide synthesis and an example is the extracellular peptidase from *Halobacterium halobium* which was exploited for efficient peptide synthesis in Water-*N*′-*N*′-dimethylformamide [16].

Given the promising potential for the biotechnological applications of these organisms, this work presents the recent advance in the knowledge of hydrolases from marine extremophiles and their biotechnological potential. Peptidases, lipases, amylases, cellulases and other cell wall-degrading hydrolases in halophiles, thermophiles, psychrophiles and piezophiles will be the main focus of this review.

## 2. Extremophiles

**Figure 1:**
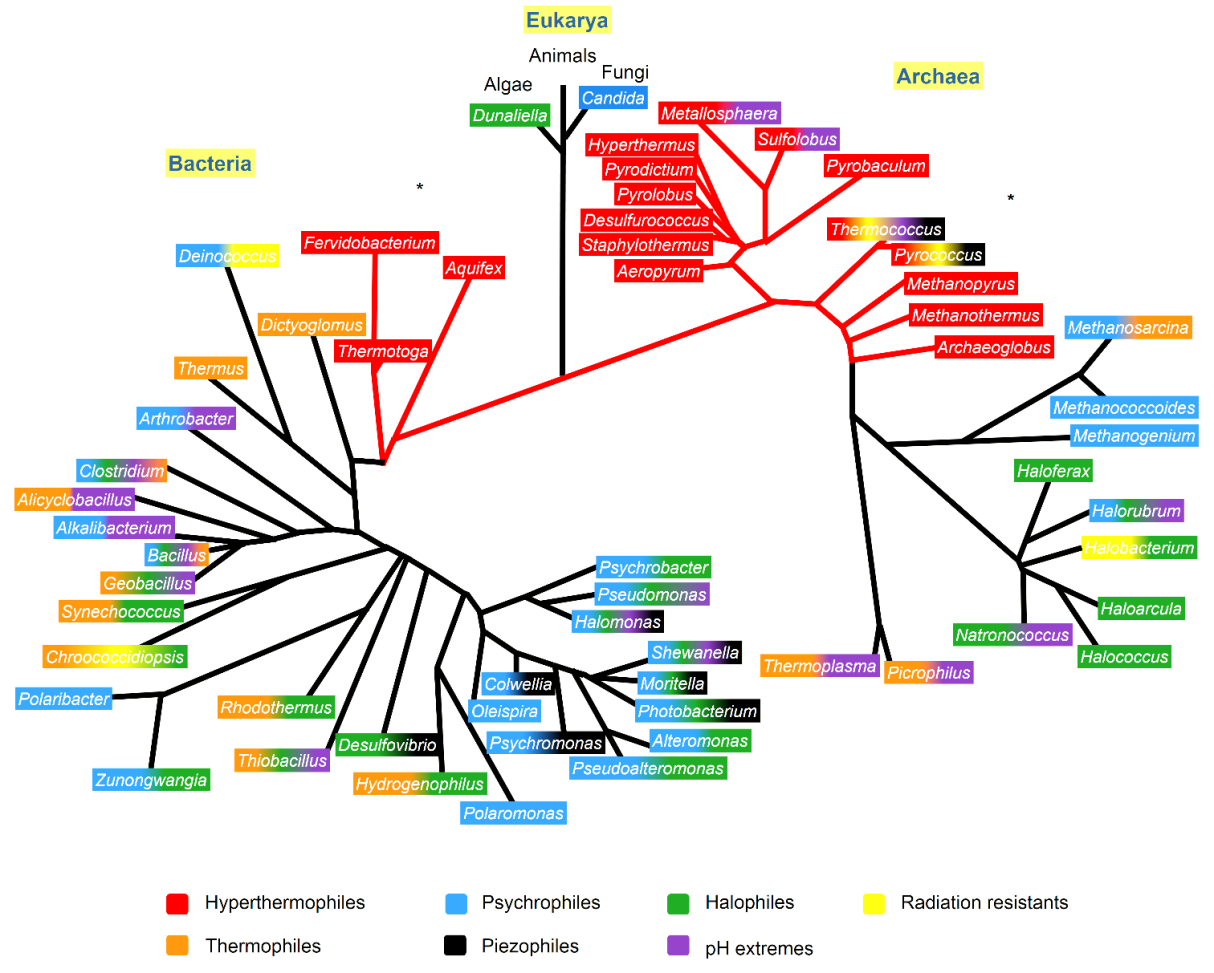
Phylogenetic tree showing the extremophiles and the resistant characteristics that appear in at least one species of each genera, identified with the color code. The phylogenetic tree was based on Woese *et al.* [28], Lang *et al.* [29] and Dereeper *et al.* [30]. The * indicates the phylogenetic branch that were according to Lang *et al.* [29].

Extremophile organisms are classified as living organisms able to survive and proliferate in environments with extreme physical (temperature, pressure, radiation) and geochemical parameters (salinity, pH, redox potential). Polyextremophile microorganisms are those that can survive in more than one of these extreme conditions. The vast majority of extremophile organisms belong to the prokaryotes, and are therefore, microorganisms belonging to the Archaea and Bacteria domains [17,18]. A phylogenetic tree showing the microorganisms of different genera and their extremophilic characteristics is presented in Figure 1.

Extremophile microorganisms are classified according to the extreme environments in which they grow and the major types are summarized in Table 1. Different structural and metabolic characteristics are acquired by these organisms so that they can survive in these environments [19,20]. Because of the ability to withstand extreme situations, possible industrial applications of extremophiles have been widely investigated [21,22,23,24,25].

One of the most well known applications of an extremophile is the DNA polymerase of the extremophile *Thermus aquaticus* (Taq polymerase) [26] which is widely used in the polymerase chain reaction (PCR). The stability and enzymatic activity of extremophiles and their extremozymes are useful alternatives to conventional biotechnological processes [27].

**Table 1:** Extremophile microorganisms and their environments (adapted from Horikoshi and Bull [17]).

Extremophile Microorganism	Favorable Environment to Growth
Acidophile	Optimum pH for growth—Below 3
Alkaliphile	Optimum pH for growth—Above 10
Halophile	Requires at least 1M salt for growth
Hyperthermophile	Optimum growth at temperatures above 80 °C
Thermophile	Grows at temperatures between 60 °C and 85 °C
Eurypsychrophile (psychrotolerant)	Grows at temperatures above 25 °C, but also grow bellow 15 °C
Stenopsychrophile (psychrophile)	Grows at temperatures between 10 °C and 20 °C
Piezophile	Grows under high pressure—Above 400 atm (40 MPa)
Endolithic	Grows inside rocks
Hipolith	Grows on rocks and cold deserts
Oligotroph	Able to grow in environments of scarce nutrients
Radioresistant	Tolerance to high doses of radiation
Metallotolerant	Tolerance to high levels of heavy metals
Toxitolerant	Tolerates high concentrations of toxic agents (eg. Organic solvents)
Xerophile	Grows in low water availability, resistant to desiccation

### 2.1. Thermophiles

The thermophile marine microorganisms include several groups such as the phototrophic bacteria (cyanobacteria, green and purple bacteria), bacteria domains (*Actinobacteria* sp., *Bacillus* sp., *Clostridium* sp., *Desulfotomaculum* sp., *Thermus* sp., *Thiobacillus* sp., fermenting bacteria, spirochetes and numerous other genres) and the archaea domains (*Pyrococcus* sp., *Sulfolobus* sp., *Thermococcus* sp., *Thermoplasma* sp. and methanogenic) [31,32,33]. The maximum temperature that hyperthermophile organisms have been observed to tolerate is around 120 °C [34,35].

Thermophiles have several mechanisms to support extreme temperatures. It is believed that the thermostability of cellular components such as ATP, amino acids, and peptides may exceed 250 °C, suggesting that the maximum temperature for life goes beyond the temperatures that have been observed until now [36,37]. The proteins of organisms adapted to extreme temperatures generally have similar three-dimensional structures of mesophilic organisms but the amino acid content is different from ordinary proteins and the number of charged residues on their surfaces is much greater than nonadapted organisms. In addition, such proteins often have shorter loops, thus preventing the occurrence of nonspecific interactions due to their increased flexibility at high temperatures [38,39].

Extreme thermophile bacteria produce thermostable proteins that can be readily crystallized to obtain stable enzymes for structural and functional studies. Proteins from hyper/thermophiles require sufficient structural rigidity to resist unfolding. This is an important feature to characterize antidrug targets. A classical instance is the bacteria *Thermus thermophilus* that was originally isolated from a thermal vent within a hot spring in Izu, Japan, and is frequently used in genetic manipulation studies. The DNA gyrase from this extremophile has been used as an antidrug target model. DNA gyrase is a type IIA topoisomerase that introduces negative supercoils into closed circular bacterial DNA using ATP hydrolysis. It is an important antibacterial target that is sensitive to the widely-used fluoroquinolone drugs [40,41].

The thermal hypothesis determines that a G:C pair and its contents are related to thermostability. This is observed for several thermophilic bacteria. *Geobacillus thermoleovorans* CCB US3UF5 is a thermophilic bacterium that was isolated from a hot spring in Malaysia and is a source for thermostable enzymes. The bacteria contains a circular chromosome of 3,596,620 bp with a mean G:C content of 52.3% [42]. However, a study reported a comparative analyses of G:C composition and optimal growth temperature with 100 prokaryote genomes (Archaea and Bacteria domains) that failed to demonstrate this correlation (G:C/thermostability). Moreover the authors related that the G:C content of structural RNA (16S and 23S) is strongly correlated with optimal temperature and it is higher at high temperatures [43]. An increased number of disulfide bonds improve stability within thermophilic proteins and play a role in preventing the alteration of the quaternary structure [44].

Ether lipids are always present in thermophile archaea without exception, but mesophilic archaea also have ether lipids. The presence of isoprenoid chains in archaea membranes is associated with two properties to maintain the thermostability of the lipid membrane: A high permeability barrier and a liquid crystalline state. Bacterial membranes only keep these states at the transition phase of temperature [45].

Accordingly, enzymes adapted to higher temperatures bring advantages to industrial processes, promoting faster reactions, high solubility of the substrate, a lower risk for contamination of the system, and also lowering the solution viscosity and increasing the miscibility of the solvent [46]. Several thermophiles and thermostable enzymes are used in the biorefinery industry, first and second generation biofuels, paper and bleaching industries [47,48,49].

### 2.2. Psychrophiles

Earth is primarily a cold marine planet: 90% of the water in the oceans has temperatures of 5 °C and 20% of the terrestrial region of the Earth is permafrost (frozen soils), glaciers and ice sheets, polar sea ice and snow covered regions [50]. These regions have cold-adapted microorganisms which have a restricted temperature range for growth. Stenopsychrophiles (formerly true psychrophiles) have an upper temperature limit of 20 °C for growth. However, the majority of isolates are eurypsychrophile (formerly psychrotolerant) and have a broader temperature range, tolerating warmer environments [51]. Psychrophile microorganisms are often found in other extremely cold environments such as deep oceans, caves, land surfaces and even in the upper atmosphere [52]. They have been described performing DNA synthesis at −20 °C and active metabolism at −25 °C [53,54].

Various stenopsychrophiles isolated from Antarctic have been studied; for example the genera Arthrobacter, Colwellia, Gelidibacter, Glaciecola, Halobacillus, Halomonas, Hyphomonas, Marinobacter, Planococcus, Pseudoalteromonas, Pseudomonas, Psychrobacter, Psychroflexus, Psychroserpens, Shewanella and Sphingomona. Methanogens, members of Archaea, are the only group known to have individual species able to grow in a very wide temperature range from subzero to 122 °C [55,56].

The adaptations and mechanisms related to life in icy environments include responses to cold shock and RNA chaperones. Cold shock proteins (CSPs) act as cold-adaptive proteins in psychrophiles. They are small proteins that bind to RNA to preserve its single-stranded conformation and contain a nucleic-acid-binding domain, known as the cold shock domain (CSD), and also they have additional roles besides serving as RNA chaperones [57,58]. Small RNA-binding proteins (RBPs) can facilitate cold adaptation but together with the CSPs they have other functions in bacteria. RNA helicases are regulated during cold growth and are capable of unwinding secondary structures in an ATP-dependent manner in some psychrophiles [58]. The presence of dihydrouridine can enhance tRNA flexibility and is elevated in some psychrophilic bacteria and archaea [59].

Other factors involved in cold adaptation are the production of secondary cold active metabolites, enzymes that are activated and induced by cold, antifreeze proteins, and the production of pigments and membrane fluidity [60,61,62]. From the structural point of view, the proteins of these organisms have a higher content of α-helix relative to the β-sheets, which is considered to be an important factor to maintain flexibility even at low temperatures [63]. Also the cytoplasmic membranes of these microorganisms contain a higher proportion of unsaturated fatty acids (52%) compared to mesophilic (37%) and thermophilic (10%) organisms, favoring the maintenance of the semi-fluid state of membranes [64]. Marine psychrophiles participate in biogeochemical cycling, polar food web and produce a wide variety of enzymes including amylases, cellulases, peptidases, lipases, xylanases and other classes of enzymes [50,65]. High rates of catalysis at low temperatures are generally achieved by the flexible structure and low stability of cold-active enzymes. The most common adaptive feature of cold-active enzymes is a reaction rate (*k_cat_*) that is largely independent of temperature [56,66].

Furthermore, enzymes adapted to lower temperatures allow efficient production at low cost, save energy, and are important in thermal protection, resulting in improvements in the quality of various products [67]. Enzymes suited to low temperatures are used in the food, cosmetics, pharmaceutical, and biofuels industries; also they are applied to substances for molecular biology, nanotechnology, in the manufacturing of household detergents, in the cleaning of animal wastes, with peptidases for cleaning contact lenses and pectinases to extract and clear fruit juices [67,68]. A clear example of the benefit generated by the industrial application of cold adapted microorganisms is the use in the hydrolysis of lactose at low temperatures in the process of milk storage. This bioprocess has enabled the production of milk for patients with milk intolerance and is now being patented for use on a large scale by Nutrilab NV (Bekkevoort, Belgium) [69].

### 2.3. Halophiles

Halophiles are microorganisms that require salt (NaCl) for growth, and they can be found in lakes, oceans, salt pans or salt marshes. Moreover about 25% of the available land on Earth is in the form of saline deposits [70]. According to the optimal salt concentration for growth, they are classified in three categories: (i) extreme halophile—Grows in an environment with 3.4–5.1 M (20% to 30%) NaCl; (ii) moderate halophile—Grows in an environment with 0.85–3.4 M (3% to 25%) NaCl; and (iii) slightly halophile—Grows in an environment with 0.2–0.85 M (1% to 5%) NaCl [31]. Halotolerant microorganisms do not show an absolute requirement for salt to grow but grow well in high salt concentrations [71].

Members of the family *Halobacteriaceae* have been isolated from different habitats including alkaline and salt lakes, marine salterns, the Dead Sea and saline soils. Deep hypersaline anoxic basins (DHABs) or deep-sea hypersaline anoxic lakes (DHALs) are extreme habitats that have been discovered on the sea floor in different oceanic regions, such as the Gulf of Mexico, the Red Sea and the Eastern Mediterranean Sea. DHABs are composed by dissolution of evaporitic deposits, entrapped in the sea floor, forming a very stable brine and sharply stratified in water columns, a chemocline. The *b*rines enclosed in these basins are characterized by hypersalinity, 5–10 times higher than seawater, a lack of oxygen and highly reducing conditions, high pressure (around 350 atm–35 MPa) and absence of light. These physicochemical parameters make the DHABs one of the most extreme environments of the planet and they have also ensured that those habitats have been maintained isolated for thousands of years [11,72,73].

Since the discovery of the first Mediterranean DHAB named *Tyro* in 1983, six others have been unveiled: *l*’*Atalante*, *Bannock*, *Discovery*, *Medee*, *Thetis*, and *Urania* [74], and these habitats are a source of anaerobic halophilic microorganisms. Prokaryotes from the Bacteria and Archaea domains belonging to new taxonomic lineages were discovered in high abundance in DHABs by 16S rRNA libraries and fluorescent *in situ* hybridization (FISH). Microbiologically DHABs of the Eastern Mediterranean are the most studied. *Halorhabdus utahensis* constitutes 33% of the total archaeal community and tolerates up to 0.8 M MgCl_2_ [75]. In the Mediterranean Ridge, a DHAL named *Kryos* has been identified. This lake is filled with MgCl_2_-rich, athalassohaline brine (salinity > 470 practical salinity units). Two groups of halophilic euryarchaeal divisions (MSBL1 and HC1) account for ~85% of the rRNA-containing archaeal clones analyzed in the 2.27–3.03 M MgCl_2_ layer [74]. An earlier study assumed that 2.3 M of MgCl_2_ was the upper limit of concentration for life to survive [76], despite the fact that halophilic archaea have been identified in deep hypersaline anoxic basins composed of saturating concentrations of MgCl_2_ [73,77]. Antunes *et al.* [78] recently published a long list of microorganisms in a microbiological review of these unique deep-sea anoxic environments.

In addition, some halophiles are thermostable and tolerant to a wide range of pH. The metabolic diversity of halophiles is widely spread, comprising the anoxic phototrophic, aerobic heterotrophic, fermenter, denitrifying, sulfate reducers and methanogenic organisms [79].

Halophiles have developed different adaptive strategies to support the osmotic pressure induced by the high NaCl concentrations in the environments they inhabit. Some extremely halophilic bacteria accumulate inorganic ions (K^+^, Na^+^, Cl^−^) in the cytoplasm, which is a type of “salt-in” strategy to balance the osmotic pressure of the environment, and they have also developed specific proteins that are stable and active in the presence of salts [80,81,82]. Also, the halophile organisms contain enzymes that maintain their activity at high salt concentrations, alkaline pH and high temperatures [71]. Most proteins and enzymes denature when suspended in high salt concentrations. Halophilic proteins bind significant amounts of salt and water. This characteristic is dependent on the number of acidic amino acids on the surface of the protein.

The function of electrostatic interactions in the stability and folding of halophilic proteins has been investigated and is an important determinant of haloadaptation. The intracellular K^+^ ions of haloarchaea have been found to be extremely high, near to 5 M [83]. Based on the comparative analyses of halophile and non-halophile proteomes, the amino acid composition of halophilic enzymes is in general characterized by an abundant content of acidic amino acid, a high proportion of aspartic and glutamic acids, a low frequency of lysine, and a high occurrence of amino acids with a low hydrophobic character. Structural analyses between halophilic and mesophilic proteins reveal that the major differences are concentrated on the surface of the protein. These characteristics allow cooperation with electrostatic interactions and the presence of a higher number of salt bridges [84]. The stability of the enzymes depends on the negative charge on the surface of the protein due to acidic amino acids, the hydrophobic groups in the presence of high salt concentrations and the hydration of the protein surface due to carboxylic groups present in aspartic and glutamic acids. In addition, negative surface charges are thought to be important for the solvation of halophilic proteins, to prevent denaturation, aggregation and precipitation [85,86].

Moderate halophiles use other haloadaptations based on biosynthesis and/or accumulation in the cytoplasm of high amounts of specific organic osmolytes, which function as osmoprotectants, providing osmotic balance and maintaining low intracellular salt concentrations without interfering in the normal metabolism of the cell. The osmolyte could be obtained by direct uptake from the environment [85]. These solutes can act as stabilizers for biological structures and allow the cells to adapt not only to salts but also to heat, desiccation, cold or even freezing conditions [87]. Many halophilic bacteria accumulate ectoine or hydroxyectoine as the predominant compatible solutes. Other types of osmolyte include glycine, betaine and other neutral glycerols [71].

One of the adaptation mechanism developed is the lipid composition. Structural adaptations have been observed in the S-layers of halophiles. The extreme halophile contains sulfated glucuronic acid residues and a higher degree of glycosylation, leading to an increased density in surface charges. This characteristic demonstrates an adaptation in response to the higher salt concentrations experienced by *Halobacterium salinarum.* Moreover, in *Haloarchaea*, some S-layer glycoproteins are enriched in acidic residues [88].

The halophiles have been used in biodegradation of organic pollutants, in desalinization of wastewater, in nanotechnology, in production of biopolymers and as osmoprotectors [89,90]. The halotolerance of hydrolases derived from halophilic bacteria can be exploited wherever enzymatic transformations are required to function under physical and chemical conditions, such as in the presence of organic solvents and extremes in temperature and salt content. Many halophiles can secrete extracellular hydrolytic enzymes, such as amylases, lipases, peptidases, xylanases and cellulases that are thermostable and adaptable to a wide range of pH [80].

### 2.4. Piezophiles

High hydrostatic pressure is one of the physical parameters in deep-ocean environments and it plays a selective role in the distribution of life on the planet. The oceans, which have an average depth of 3800 m and an average pressure of 380 atmosphere (atm) or 38 MPa, make up ~95% of the biosphere. In the deepest parts of the oceans, pressures of 700 to 1100 atm (70 to 110 MPa) prevent the growth of most microorganisms. Moreover, the temperature in the deep-sea is typically within the 1–3 °C range. However, there are hydrothermal vent habitats where high pressures and high temperatures are found, and in these regions marine microbes might be exposed to temperatures and pressures ranging from 1–300 °C and 1–1100 atm (0.1–110 MPa), respectively [91].

The effects of high hydrostatic pressure on microbial metabolisms occurs in the cellular structures and cellular processes such as cell division and motility [92]. Microorganisms called piezophiles (previously named barophile), such as deep-sea bacteria or archaea, live in high pressure environments and are of interest to various sectors of biotechnology [93]. The Mariana Trench is the deepest part of the ocean found on the planet; it has a maximum depth of 11 km and a pressure of 1100 atm (110 MPa). This extreme habitat harbors organisms that can grow in standard pressure and temperature and strict piezophiles, like *Moritella yayanosii* and *Shewanella benthica*, that have pressure growth conditions of between 700 and 800 atm (70 ~80 MPa), but not less than 500 atm (50 MPa) [18,94]. 

Microorganisms which possess optimal growth rates at pressures above atmospheric pressure are classified as piezophilic; and as piezotolerant those that grow at high pressure, as well as at atmospheric pressure but they do not have optimal growth rates at pressures above one [91]. Bacterial piezophiles are mainly psychrophiles belonging to five genera of γ-proteobacteria, *Photobacterium*, *Shewanella*, *Colwellia*, *Psychromonas* and *Moritella*, while piezophilic Archaea are mostly (hyper)thermophiles from *Thermococcales* [95].

The physiological adaptations required for growth under these extreme conditions are substantial and involve a combination of modifications of gene structure and regulation. The adaptation mechanisms of piezophiles are under investigation. Whether piezophilic adaptation requires the modification of a few genes, or metabolic pathways, or a more profound reorganization of the genome has not yet been fully elucidated [95]. As with psychrophiles, piezophiles contain lipids with highly unsaturated fatty acids [96]. Other adaptation mechanisms against the high pressures include reduction of cell division, modification of membrane and transport proteins and accumulation of osmolytes, which stabilize the proteins [94,97,98]. The occurrence of elongated helices in the 16S rRNA genes to increase adaptation to growth at elevated pressure has also been described [99].

### 2.5. Polyextremophiles

Microorganisms in their natural habitats are thought to experience stress during their life cycle. Many extremophiles inhabit environments with more than one extreme parameter, for example, extremophiles that thrive in the depth of the oceans or close to hot springs. In the first situation, if the extremophiles are found in ocean mud, they could be piezophiles or psychrophiles, but if they were found close to a hydrothermal vent, they could be piezophiles, thermophiles or acidophiles, due to the minerals released in the chimney, or even, if they were found in DHABs, they could be piezophiles, psychrophiles or halophiles.

In an effort to provide a comprehensive look at the extremes of temperature and pH, Capece *et al.* [100] tabulated over 200 extremophile species found in the literature. They are called thermoacidophiles, thermoalkaliphiles, psychroacidophiles and psychroalkaliphiles. Since membrane fluidity decreases at low temperatures, the lower permeability to protons (H^+^) becomes an advantage for acidophiles and alkaliphiles. The pH homeostasis is controlled by H^+^ movements across the membrane. The presence of psychroacidophiles and psychroalkaliphiles has not yet been found in nature; only the presence of psychrotolerant alkaliphiles, such as *Alkalibacterium psychrotolerans* has been observed [100,101].

However, the presence of thermoacidophiles is well documented. High temperatures increase the permeability to H^+^ resulting in a lethal cytoplasmic acidification [102]. Modifications in RNA codon thermostability and a neutral surface charge in proteins prevent the occurrence of an acid hydrolysis. The existence of thermoacidophiles over 100 °C has not yet been observed [100,103]. The survival of thermoalkaliphiles is thought to be related more to the buffering capacity to maintain a stable intracellular cytoplasm than the maintenance of a bioenergetic gradient [100,102,104]. 

High salt concentrations allied to temperature extremes have been observed in psychrohalophiles and thermohalophiles. Many environments in polar sea ice are cold brine solutions, thus some degree of halophily is typical in most psychrophiles. Indeed, cold adaptation and salt adaptation have common approaches [100]. Thermohalophiles are rare. Their uncharged proteins became extremely unstable in hypersaline solutions, and are denatured by solvents at high temperatures and by decreasing the electrostatic interactions required to maintain the native folding [105,106]. *Thermococcus waiotapuensis* is the most hyper-thermohalophilic organism discovered to date, however, the basis of its biochemical stability is still unclear [107].

The correlation of temperature and pressure is also another parameter involved in extremophilic survivability. Increases in volume are favored at high temperatures but disfavored at high pressures. Thermopiezophile protein adaptations are synergistic, comprising a small surface charge with a strongly hydrophobic core. Other biochemical responses are the induction of both heat-shock and cold-shock response pathways reinforcing the synergistic reaction [100,108]. The cold-shock response is extremely important in psychropiezophiles, since they do not benefit from the synergistic temperature and pressure approach. They also incorporate monosaturated and polysaturated fatty acids to prevent membrane crystallization, which is caused by both extremes of temperature, a characteristic already incorporated in piezophiles [100,109].

Halo-acidophiles and halo-alkaliphiles are both found in nature. For halo-acidophiles, high extracellular concentrations allow a more favorable efflux of H^+^. Halo-alkaliphiles are more commonly found, because the monovalent cations of salts are essential for pH homeostasis and energetic coupling [110,111]. On other hand, for halo-alkaliphiles, the less favorable influx of H^+^, caused by high salt concentrations, leads to a lethal alkylation of the cytoplasm, and becomes a limitation to growth. Halo-alkaliphiles are able to maintain a gradient of about 1.0–1.5 units [102,112]. Finally, high pressures promote negative volume changes by the dissociation of acids and the protonation of amine groups in proteins. This leads to an acidification of the solution, creating an environment where piezo-acidophiles can be found. On the other hand, the alkalinification of the environment associated to high-pressures creates a propitious site for piezo-alkaliphiles. However, both of these organisms still need to be further investigated for systematic identification and characterization [100,113].

Polyextremophilic enzymes have been applied in the food, detergent, chemical, pulp and paper industries. A thermo-alkali-stable enzyme from *Bacillus halodurans* TSEV1 has applicability in pre bleaching of paper pulp and recently has been expressed in *Pichia pastoris* for the production of oligosaccharides [114,115]. Another strain of *B. halodurans* PPKS-2 produced an alkaliphilic, halotolerant, detergent and thermostable mannanase. This strain grows in agro wastes and can be applied for mannanase production on an industrial scale for detergent and pulp and paper bleaching [116]. The Antarctic cold-adapted halophilic Archeon *Halorubrum lacusprofundi* produces a recombinant polyextremophilic enzyme that is active in cold temperatures, high salinity and is stable in aqueous-organic mixed solvents. This enzyme is suitable for applications in synthetic chemistry [117].

## 3. Biocatalysis: Bioengineering and Other Strategies

The application of hydrolases in industrial processes sometimes fails due to the lack of robustness, stability and undesirable properties [5]. Recent experimental advances, associated to novel bioinformatic tools and protein engineering, has allowed the development of more efficient hydrolases for industrial purposes [46,118,119]. Since the introduction of site-directed mutagenesis and other integrated modern techniques such as synthetic biology and system biology, it is now possible to modify the characteristics of these enzymes, such as enhancing their stability and specificity [120,121].

Anaerobic, extremophilic and marine bacteria are a source of enzymes with superior chances of success in biotechnological processes. A great deal of laboratory effort has been concentrated on their production and characterization. Furthermore, the design of novel enzymes as well as molecular approaches such as enzyme evolution and metagenomic approaches can be used to identify and develop novel biocatalysts from uncultured bacteria—A treasure-trove of unknown proteins. Some characteristics of extremophiles are important for obtaining recombinants with specific properties. The advantage of psychrophile enzymes is their reduced energy consumption. Thus, psychrophiles could be used in the production of thermolabile proteins for genetic engineering [60].

Stabilization of hydrolytic enzymes is of interest due their potential applications in the medical, chemical and pharmaceutical industries. Methods to stabilize proteins include protein engineering or chemical modifications [122,123]. In the past two decades, protein engineering has become a powerful means to alter or improve enzymatic catalysis [124,125], and is divided into two methods: (1) directed evolution—A random mutagenesis is applied to a protein; and (2) rational protein design—Knowledge of the structure and function of the protein is necessary in order to change its characteristics. These methods have been successfully applied to increase protein activity, selectivity or thermostability [126]. The protein engineering technique is helping to improve the production of chemicals, such as optically pure tertiary alcohols and drugs, like the profen family (NSAIDs) [127,128]. Directed evolution has been reported to be laborious and costly, however it does provide the means for selecting mutants with improved properties [122,123]. Allying chemical modifications to recombinant DNA technology can generate improvements in enzyme stability and efficiency, preventing changes in the active-site and in targeting enzyme surface groups [129,130].

Mutations could be induced by physical and chemical methods such as UV-irradiation, γ-rays, fast neutron irradiation, nitrosoguanidine (NTG), diethyl sulfate and nitrous acid which have been applied to breed lipase-producing microorganisms. With these simple methods, the lipase yield can be improved by 1 to 10-fold; however, the low positive mutation rate, the long periodicity and the laborious screening has limited its widespread use [131].

Enhancing the stability of extremophilic enzymes is very beneficial because this could maintain the high activity at low/high physical-chemical parameters for prolonged periods of time, a characteristic that can be exploited in many industrial processes. However, basic issues involved in the stabilization mechanisms need to be analysed before any modifications can be made. These issues include the type and size of the protein, the structure and size of the modifying reagent, the chemical reactions involved in the modification procedures and the conditions of such modifications [122,123].

Siddiqui *et al.* [132] demonstrated that chemical modifications could be useful in providing details of structure, function and stability of proteins, turning them into potential guides for future target studies, including an attempt to convert mesophilic enzymes into cold-adapted ones. Tadeo *et al.* [84] demonstrated that it is possible to decrease the salt dependence of a halophilic protein to the level of a mesophilic one, and engineering the protein in an inverse form, from mesophilic to an obligate halophilic form, suggesting that the halophilicity is related exclusively to surface residues.

On the other hand, searching for enzymes that already exist in nature could be faster and more straightforward than the engineering routes. Metagenomic techniques could be a powerful tool for making such discoveries, by trying to find novel genes and enzymes from uncultured microorganisms [133]. Metagenomic analysis can be either sequence driven, for example, accessing the 16S rRNA, recA or other conserved sequences, or function driven, expressing features such as a specific enzyme activity or antibiotic production [134]. The 16S rRNA metagenomic studies provide an enormous diversity of free-living marine microorganisms from different environments and include the discovery of new enzymes of microbial origin [135]. A list of marine enzymes discovered by metagenomics can be found in Kennedy *et al.* [136].

Some advantages can be applied to functional metagenomics over the sequence methods. The functional approach recognizes the genes by their function, rather than their sequences, avoiding incorrect annotations or similar sequences of gene products with multiple functions. When you search for novel functions or a complete new class of genes, this could be crucial [136]. Currently platform databases, such as KEGG (Kyoto Encyclopedia of Genes and Genomes) and GenomeNet, are helping in the interpretation of a large amount of data generated by metagenomic output analyses. A large number of bioinformatic tools are now available for Data Mining of metagenomic sequences, based on a series of features [136,137]. Combining different approaches, like activity-based mining with tailoring of robust and selective enzymes, would give a biotechnological process a chance to take over from chemical catalysis, transforming industrial chemistry into a more eco-friendly process [138].

Currently all these techniques are powerful tools for biocatalysis. Miyake *et al.* [139] constructed a recombinant protein expression system using the psychrophilic bacterium *Shewanella* sp., isolated from Antarctic seawater. This bacterium grows at temperatures close to 0 °C. The enzyme produces β-lactamase reaching 91 mg/liter of culture at 4 °C and 139 mg/liter of culture at 18 °C. In another study, mesophilic enzymes were expressed in psychrophilic microorganisms for efficient 3-hydroxypropionaldehyde production from glycerol, using *Shewanella livingstonensis* Ac10 as a selected host [140]. A heat-sensitive esterase and two chaperones (Cpn60 and Cpn10) from a psychrophilic bacterium, *Oleispira antarctica* RB-8 T were simultaneously expressed in *E. coli*. The resulting enzyme from the recombinant strains was more active (180 fold) than the *E. coli* strain grown at 37 °C and was also active at low temperatures [141].

Other more suitable techniques can be applied in the search for novel enzymes from rare microorganisms, such as single cell genomics, metatranscriptomics and metaproteomics. The single cell genomics allow the study of the entire biochemical process of a single uncultured cell; the metatranscriptomics can access only the transcriptionally active genes in their specific population; and the metaproteomics can analyze the enzymes directly involved in a particular biochemical pathway [136].

Systems Biology is another integrated strategy used in industrial biotechnology. A quantitative analysis of biological systems is carried out using a mathematical model via computer simulation. Cellular metabolisms are analyzed and optimized for application in the development of strains and bioprocesses [121]. “Omics” technologies have been employed in studies such as: Transcriptome (genome-wide study of mRNA expression levels), proteome (analysis of structure and function of proteins and their interactions), metabolome (measurement of all metabolites to access the complete metabolic response to a stimulus), fluxome analysis (metabolic flux) and also in advanced mathematical modeling tools, such as genome-scale metabolic modeling [5,121].

Synthetic Biology is a field that has been growing in the last few years and is in many ways related to genetic engineering. It is an integrated and interdisciplinary theme including bioinformatics, microbiology, molecular biology, systems biology and biology in order to design and construct new biologic functions and systems not found in nature or improving certain functions through the creation of a synthetic genome [120].

## 4. Hydrolases and Their Biotechnological Potential

Currently more than 500 products are produced using enzymes and about 150 industrial processes benefit from the use of enzymes or catalysts from microorganisms. Moreover, more than 3000 enzymes are known and approximately 65% are hydrolases used in the detergent, textile, pulp, paper and starch industries and almost 25% of these are used for food processing [142,143]. Studies show that the diversity of extremophile microorganisms may be higher than we think [31,144]. However, the characterization and use of such a diversity of enzymes becomes complicated due to the difficulty of isolating and growing these microorganisms [142].

By definition, hydrolases are enzymes that catalyze reactions with the substrate through the hydrolysis of chemical bonds. Hydrolases are enzymes classified as Class 3 (EC 3) by the Nomenclature Committee of the International Union of Biochemistry and Molecular Biology (NC-IUBMB) that keeps an updated list of all the enzymes described in a database, available at ExplorEnz [145].

## 5. Amylases from Marine Extremophiles

Starch comprises an abundant source of available energy and is present in almost all higher plants. Starch is a polymer composed of glucose molecules that form a so-called straight-chain amylose via the α 1–4 linked type. The association of α 1–4 with the α 1–6 type branches creates amylopectin, the largest part of the starch molecule [146].

**Figure 2:**
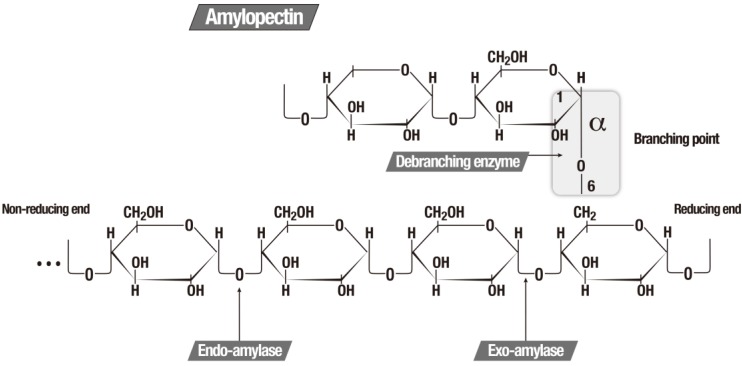
The three major amylolytic enzymes [5].

Amylases are enzymes, which hydrolyze the starch molecules to glucose monomers and can be classified according to the specificity of the substrate in which they operate, as showed in Table 2 [5,147]. Figure 2 illustrates the function of the three major amylolytic enzymes.

The amylolytic enzymes are one of the most interesting enzymes for industrial processes. The α-amylase of different species of the genus *Bacillus* are the amylases that are the most applied in biotechnological processes, because of their thermophilic properties and high conversion rates [148]. The research for extremozymes and their special characteristics for industrial processes has been expanded [149], and over the years several stable amylases from thermophiles, psychrophiles, alkaliphiles, acidophiles and halophiles have been reported [150,151,152]. They are found in different genera of marine extremophile archaea and bacteria from the surface to deep sea locations, and include *Desulfurococcus* sp., *Pseudoalteromonas* sp., *Pyrococcus* sp., *Rhodothermus* sp. and *Thermococcus* sp. [153,154,155,156]. *Geobacillus* sp. is an isolate from a geothermal vent, and has a remarkable alpha-amylase stability between 80 °C to 140 °C [157].

**Table 2:** Classification of Amylases.

		Enzyme	Classification	Cleavage	Product
**Amylases**	**Endoamylase**	α-amylases	EC 3.2.1.1	Internal α-1,4	Dextrins
	**Exoamylases**	β-amylase or maltase	EC 3.2.1.2	Outer regions of α-1,4	Maltose
		Glucoamylase	EC3.2.1.3		β-cyclodextrin and glucose
		α-glucosidase	EC 3.2.1.20		
	**Debranching**	Pullulanases	EC 3.2.1.41	α-1,6 linkages	Maltotriose
		Isoamylases	EC 3.2.1.68	Pullulan	Malto-oligosaccharides
		Dextrinases	EC 3.2.1.142	α-1,6 linkages	Maltose

Several industrial processes can be performed with the use of amylases. The endoamylases, which have optimum activity for temperatures between 70 °C and 95 °C have been applied in the production of ethanol from corn starch, rice or as a pretreatment for forming sugars (saccharification) for fermentation [158,159]. Amylases are also used in the food industry for the production of glucose syrups, fructose and maltose, reduced viscosity of syrups, reducing the turbidity of juices and also for alcohol fermentation. They are also involved in the textile, papermaking, detergent, chemical, pharmaceutical and petroleum industries [5,160,161].

Thermostable amylases are important for starch hydrolysis under high temperatures, accelerating the reactions and reducing possible contaminations [148]. Many companies sell different thermostable and broad-range pH operating enzymes. This is the case of Fuelzyme^®^—Verenium Corporation (San Diego, CA, USA), an alpha-amylase which was originated from *Thermococcus* sp. isolated from a deep-sea hydrothermal vent. Fuelzyme^®^ is applied to mash liquefaction during ethanol production, releasing dextrins and oligosaccharides with better solubility and with low molecular weight. It operates in a pH range of 4.0–6.5 and temperatures above 110 °C [162]. However, Fuelzyme^®^ and Spezyme^®^ (DuPont-Genencor Science) are only used for biofuel production. The use of a blend of these commercially available amylases and others from *Bacillus* sp. has been suggested to make industrial starch processing more efficient, and suitable for downstream applications [163].

Pullulanases are commonly associated to glucoamylases for the saccharification of starch, and there is a growing demand in industry for them [164]. Marine sources have been naturally appointed as providers of such enzymes [165]. Thermostable pullulanases from type II (amylopullulanases) are being used in the process of starch liquefaction and saccharification combined [166,167]. The hyperthermophilic archaea *Staphylothermus marinus*, an isolate from the deep-sea associated with geothermal activity or hydrothermal vents, presents an optimum growth at 98 °C. A new thermostable amylopullulanase of the glycosyl hydrolase family from *S. marinus*, has recently been described with degradation activity towards pullulan and cyclodextrin at 105 °C [168]. One of the most thermostable and thermo-active pullulanases type II was described for *Pyrococcus furiosus* and *Thermococcus litoralis*, with activity ranging from 130 °C to 140 °C in the presence of 5 mM Ca^++^ [169].

On the other hand, cold adapted amylases also have their uses in the detergents, textile and food industries, due to their considerable energy savings and reduction of bacterial contamination [150]. The most studied cold active alpha-amylase originates from *Alteromonas haloplanctis*, which is synthesized at 0–2 °C and has been successfully expressed in *Escherichia coli* [170]. The alpha-amylase from *Pseudoalteromonas haloplanktis* (AHA) (former *Alteromonas haloplanctis*) showed 80% of initial activity at 4.5 M of NaCl at 10 °C [171]. Structural features in AHA have been studied through site-directed mutagenesis and chemical modification, including a modification performed that provides support for the importance of arginine residues, instead of lysine, which enhances the enzyme thermostability to cold adaptation; however, it decreases their activity [132]. Recently, *Zunongwangia profunda* was isolated from the deep-sea and presented a cold adapted and salt tolerant alpha-amylase, one of the very few alpha-amylases that can tolerate both cold and salt conditions [172]. An alpha-amylase was found in an isolate from the genus *Bacillus* on a marine salt farm, with a hyperthermostable enzyme acting at 110 °C as optimum operating temperature [173]. The *Halomonas* sp. strain AAD21 was found to produce a halo and thermostable alpha-amylase [174]. The *Holoarcula* sp. stain S-1, produced an extracellular organic solvent-tolerant alpha-amylase that was stable and active in benzene, toluene and chloroform, with a maximal activity at 50 °C in 4.3 M of NaCl and pH 7.0 [175]. Extreme halophiles and piezophiles are not common sources of amylases. Some other examples of extremophile producers of amylases are summarized in Table 3.

**Table 3:** Extreme amylases from marine extremophiles.

Microorganism	Domain	Natural Isolation Site	Metabolism	Enzyme	Type	Reference
*Pyrococcus furiosus* (recombinant)	Archaea	Thermal marine sediments	Hyperthermophile	Amylase Endoamylase	α-amylase	[176]
*Fervidobacterium pennavorans* V5 (recombinant)	Bacteria	Hot springs, Azores islands	Hyperthermophile	Amylase debranching	Pullulanase type I	[177]
*Pseudoalteromonas haloplanktis*	Bacteria	Antarctica	Psycrophille	Amylase Endoamylas	α-amylase	[171]
*Halothermothrix orenii*	Bacteria	Tunisian salt lake	Halophile/poliextremophile	Amylase Endoamylase	α amylase	[178]
*Haloferax mediterranei*	Archaea	Saltern, Spain	Extreme halophile	Amylase Endoamylase	a-amylase	[179]

## 6. Cell Wall-Degrading Hydrolases from Marine Extremophiles

Along with starch, the cell wall is another element present in the structure of all plants and which also maintains an energy reserve, although rarely used and of difficult access. This element consists of three major polymers: Cellulose, hemicellulose and lignin. Cellulose is the most abundant macromolecule on Earth and one of the major constituents of plants, formed by β-1,4-linked glucose molecules. Due to its compactness and crystalline disposition in nature, cellulose is very resistant to hydrolysis and degradation. Hemicelluloses are non-cellulosic polysaccharides composed of complex carbohydrate polymers, where xylan and glucomannan are the main components. Lignin, with hemicellulose and pectin, fills the spaces between the cellulose fibers acting as a bonding material between the cell wall components [180].

**Figure 3:**
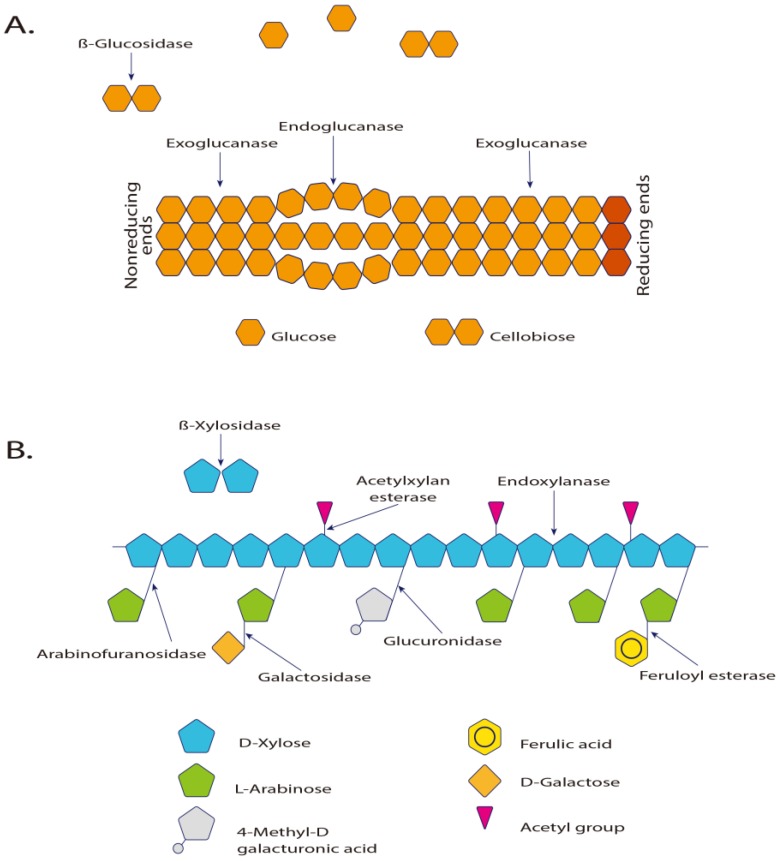
(**A**) Enzymes involved in the hydrolysis of cellulose and (**B**) in xylan hydrolysis [183]. Exo-β-1,4-glucanase and cellobiohydrolase hydrolyze the glycosidic terminals releasing cellobiose units. The β-glucosidases act directly on cellulose, hydrolyzing it to glucose. The 1,4-β-glucosidase is essential to complete the hydrolysis process of the cellulose [5]. The α-d-xylosidases are exoglycosidases that act in the non-reducing end, hydrolyzing small xylo-oligosaccharides and xylobioses, releasing xylose.

Cellulases are complex hydrolases capable of degrading insoluble cellulose polymers, present in plants, fungi and bacteria. Cellulases are among the enzymes that are the most produced for industrial purposes and it is expected that within a few years, their production will increase further, due to their use in biofuel conversion [181]. Regarding the hydrolysis of xylan, a wide variety of enzymes become necessary, and they differ in their specificity and mechanism of action [5,182]. Table 4 summarizes the classification of cellulases and xylanases. Figure 3 illustrates the dynamics of polysaccharide catalysis.

Cellulolytic microorganisms have developed complex forms of cellulolytic systems which actively hydrolyze the cellulose fibrils, and are capable of producing cellulolytic enzymes with additional functions. Furthermore, they are organized in the form of multiprotein complexes such as cellulosomes and xylanossomas [180,183,184]. The use of different vegetables such as sugarcane, corn, beets, among others for the bioenergy industry generates residues that could be reused. Biotechnological research has been stimulated to develop technologies for second-generation ethanol production from plant biomass containing lignocellulose [184,185]. Extremophile microorganisms, especially thermophilic and alkaliphiles, are widely used in lignocellulolytic processes. The thermophilic and psychrophilic cellulases have also been used in different industrial processes in the food and fermented beverages, textile, pulp and paper and animal feed industries [47,186,187]. Glycoside hydrolases are commonly found in marine thermophile-microorganisms, like *Pyrococcus* sp., *Thermococcus* sp., and *Thermotoga* sp. [156,188,189].

Beta-glycosidase from the hyperthermophilic bacteria *Thermotoga maritima*, used in transglycosylation reactions, is thermostable and resistant to a large number of proteolytic denaturants found in nature and the presence of alcohol or organic compounds stimulates its activities [190]. Thermostable xylanases are being largely used in the paper bleaching industry [191]. Xylosidases and xylanases are expressed in *Thermotoga neapolitana* during the bio-production of hydrogen, using many different carbohydrates as feedstock [192]. Recombinant xylanase is being improved to achieve more extremophilic characteristics, like *XynB* from *T. maritima*, which has been successfully expressed in *E. coli*, exhibiting thermo and alkaline stability, an attractive characteristic for bleaching kraft pulp in the paper industry [193]. Aiming for crystalline cellulose hydrolysis, a variant designed from a beta-1,4-endoglucanase (EGPh) of *P. horikoshii* exhibited stronger activity than the wild type EGPh [194].

**Table 4:** Classification of cell wall-degrading hydrolases.

		Enzyme	Classification	Cleavage	Product
**Cellulases**	**Endoglucanases**	Endo-β-1,4-glucanase	EC 3.2.1.4	Intramolecular bonds of β-1,4-glycosidic	New chain ends
	**Exoglucanases**	β-glucosidase	EC 3.2.1.21	Ends of the cellulose	Glucose or soluble cellulose
		Exo-β-1,4-glucan cellobiohydrolase	EC 3.2.1.91	Glycosidic terminals	Cellobiose
**Xylanases**		β-1,4-endoxylanase	EC 3.2.1.8	Internal glycosidic linkages along heteroxylan main skeleton	Polymerization degree of the substrate
		α-D-xylosidase	EC 3.2.1.177	Small xylo-oligosaccharides and xylobiose	Xylose

Psychrophiles are a source of these enzymes. The first cold-active and alkali-stable β-glucosidase was isolated from *Martelella mediterranea*. This enzyme retains 80% of its activity at pH 11.0 and 50% at 4 °C [195]. A deep-sea mud *Exiguobacterium oxidotolerans* also presents a cold active beta-glycosidase, maintaining 61% of its maximum activity at 10 °C and a pH range from 6.6 to 9.0 [196]. The Antarctic bacterium *P. haloplanktis CelG* gene was purified and expressed in *E. coli* for kinetic and structural optimization purposes [197]. Psychrophilic xylanases, like *TAH3a* from *P. haloplanktis* and *MSY-2* from *Flavobacterium* sp. have also been used in the baking industry [198,199]. Ideal enzymes for treating milk should work well at 4–8 °C and pH 6.7–6.8, and this is still highly desired in industrial applications. *Arthrobacter* sp. is an Antarctic isolate that produces a β-d-galactosidase capable of working at 4–8 °C, and it retains 15% of its maximum activity at 0 °C [200]. 

The slightly halophilic bacterium *Alteromonas macleodii* has been isolated from the Pacific Ocean, Mediterranean Sea, English Channel, Black Sea and Gulf of Thailand. The activities for beta-d-galactosidase, alpha-d-galactosidase and beta-d-glucosidase were found using a sample isolated from the Black Sea [201].

## 7. Marine Extremophilic Proteases

**Figure 4:**
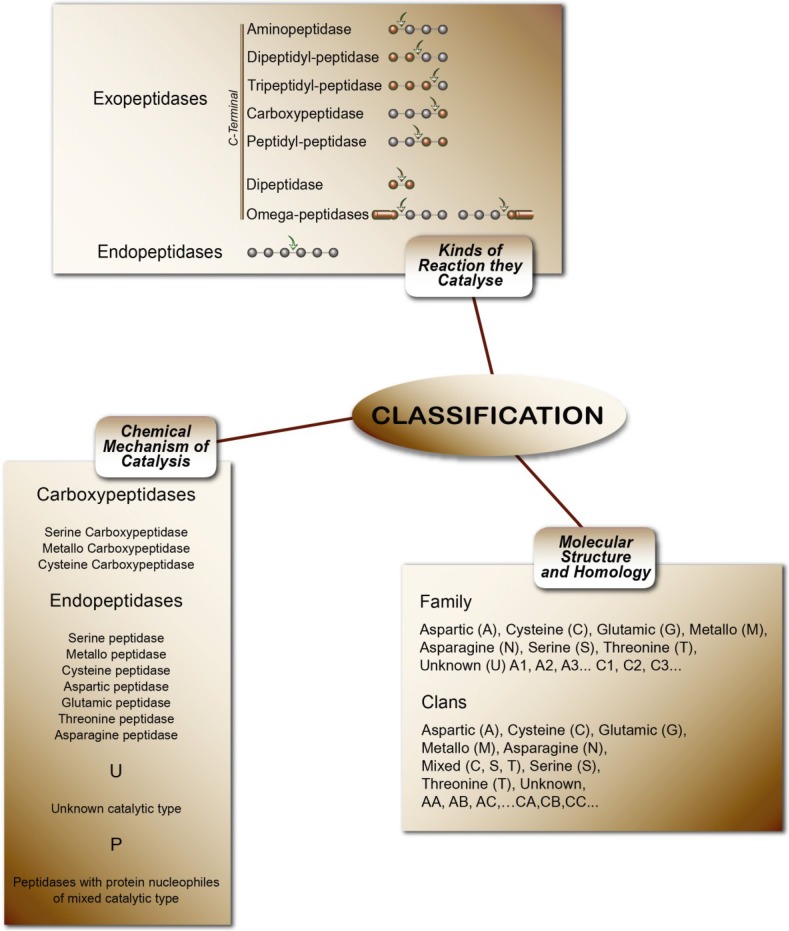
The classifications of peptidases [5].

Peptidases or proteases are proteolytic enzymes that catalyze the hydrolysis of peptide bonds on proteins or peptides. Peptidases are classified in subclass 3.4 (peptide-hydrolases). The rating uses three criteria: I—Chemical mechanism of catalysis. In this system, peptidases are classified according to their catalytic type in serine, cysteine, threonine, aspartic, glutamic, asparagine, metallo and unknown catalytic (S, C, T, A, G, N, M, and U, respectively) and P for peptidases with protein nucleophiles of mixed catalytic types. II—Type of reaction they catalyze. These are subdivided into exo-peptidases (EC 3.4.11-19), when enzymes cleave terminal amino acids, and endo-peptidases (EC 3.4.21-99), when they hydrolyze peptide bonds in the middle of the polypeptide chain. III—Molecular structure and homology. This is the most modern classification concept, based on amino acid sequences and three-dimensional structures. The peptidases are classified in families and clans. In the families, the classification of peptidases is grouped by amino acid sequence comparisons: 251 families of peptidases can be found in MEROPS Release 9. Currently the number of peptidases described and indexed in the MEROPS database exceeds more than 4000 enzymes [202]. Figure 4 summarizes the classification of peptidases.

Peptidases are enrolled in different biological processes such as regulation, localization, modulation and activities of protein interactions [203]. An important function of peptidases, is to perform posttranslational processing events, leading to the activation or inactivation of proteins, including enzymes [204]. Peptidases are also a potential drug target for microbial diseases, taking part in pathogenesis, inactivating the host immune defense mediators, in the processing of host or parasite proteins and in the digestion of host proteins. These features make peptidases very valuable to the pharmaceutical industries [5]. Other innumerous applications of peptidases are in the detergent, cosmetic, chemical and food industries [5,142].

Most peptidases from extremophilic archaea belong to the serine peptidases family, although, other families are also represented. Hyperthermophilic peptidases include serine peptidases, cysteine peptidases and the threonine-dependent proteasomes. *Pyrococus* sp. are archaeon hyperthermophiles, strictly anaerobes and obligate heterotrophs and are found in diverse habitats such as thermal marine sediments and shallow hydrothermal vents [205]. *P. furiosus* was isolated from deep-sea vents and volcanic marine mud of Italy [206]. An intracellular peptidase from *P. horikoshii* is an oligomeric cysteine peptidase [15] similar to the *P. furiosus* intracellular peptidase I [207]. In this genus, pyrolysin, a serine peptidase, metalopeptidases and ATP-dependent peptidases such as Lon A and subunits of proteasome have been characterized [208]. *Thermococcus litoralis* is a hyperthermophile archaea that is found around deep-sea hydrothermal vents as well as near shallow submarine thermal springs and oil wells. A proline dipeptidase named prolidase cleaves dipeptides having proline at the C-terminus and a nonpolar residue (Met, Leu, Val, Phe, Ala) at the amino terminus [209].

Psychrophiles are a source of peptidases and other hydrolases. The potential of the enzymes produced by them has been reviewed [65,210,211]. The diversity of extremophile microorganisms is a valuable resource in the search for new proteolytic enzymes, mainly those active at low temperatures derived from psychrophiles [69,186,212]. *S. livingstonensis* is a psychrophilic, gram-negative, isolate from Antarctic coastal areas that produces a serine alkaline peptidase [213]. *Colwellia psychrerythraea is* an obligate psychrophile, Gram-negative bacteria that can be found in cold marine environments including in Arctic and Antarctic sea-ice. The bacteria produce a peptidase of the family M1 aminopeptidase. The enzyme with a molecular mass of 71 kDa displayed an optimum temperature at 19 °C [214].

*Natrialba magadii* is an extremophile archaea that lives in alkaline hypersaline conditions (pH 9.5, 3.5 M NaCl). A Lon peptidase from this halophile was cloned and sequenced by Sastre *et al.* [215]. The ATP-dependent Lon peptidase is universally distributed in bacteria, eukaryotic organelles and archaea. In this species, like other archaea, there is a Lon B type serine peptidase, with a molecular mass of 85 kDa. *Haloferax volcanii*, a haloarchaeon isolated from the Dead Sea, also presents a Lon Peptidase. In a study by Cerletti *et al.* [216] of this species, the group demonstrated that a suboptimal cellular level of the Lon protein affected its growth rate, cell shape, cell pigmentation, lipid composition and sensitivity to various antibiotics. An extracellular serine peptidase with 45 kDa was characterized in the same species by Gimenez *et al.* [217]. *Natrialba asiatica*, which was isolated from a beach in Japan, *and H. mediterranei* isolated from seawater evaporation ponds near Alicante, Spain produced a halolysin, a serine peptidase [218]. Other extracellular and intracellular peptidase classes including metalopeptidases, and 20S proteasomes/threonine peptidases are also found in the group (for review see De Castro *et al.* [219]). Table 5 summarizes other peptidases found in marine extremophiles.

Halophilic microorganisms present some advantages in fermentation processes that occur in the presence of salt. The high salt tolerance of extreme halophiles enables their cultivation under non-sterile and thus cost-reducing conditions [220]. Some peptidases such as bromelain, papain, and pepsin have been used as biocatalysts of protein hydrolysis in fish sauce fermentation. These peptidases are not stable in high salt concentration. In contrast, peptidases from extreme halophiles require salt to carry out their activities. In addition, halophilic peptidases have a wide application in processing activities for the food, leather and detergent industries [220,221].

The *Deinococcus* genus has been isolated from a variety of habitats including Antarctica, water and activated sludge, hot springs and aquifers [222,223,224]. The fresh water radioactive site isolate, *Deinococcus aquiradiocola*, proved to be a proteolytic, capable of hydrolyzing gelatin and casein [225]. *Deinococcus indicus*, an arsenic-resistant bacterium isolated from an aquifer, has been positively characterized for casein and gelatin hydrolysis [226]. Some species have been isolated from marine habitats such as *Deinococcus geothermalis* from deep-ocean subsurfaces [227,228]. Pietrow *et al.* [229] described the production of the thermo-alkali-stable peptidase from *D. geothermalis*. The peptidase presented stability at 60 °C and pH 9.0; these properties are appropriate for the detergent industry.

**Table 5:** Peptidases from marine extremophiles.

Peptidase	Extremophile	Habitats	Reference
Serine peptidase	*Fervidobacterium pennavorans*	Hot spring	[230]
	*Sulfolobus solfataricus*	Volcanic hot spring	[231]
	*Thermococcus litoralis*	Deep-sea hydrothermal vent Shallow submarine thermal springs and oil wells	[232]
	*Thermoanaerobacter yonseiensis*	Geothermal hot stream at Sileri on Java island, Indonesia	[34,233]
Cysteine peptidase	*Aciduliprofundum boonei*	*Marine* hydrothermal vent	[234]
	*Haloferax volcanii*	Dead Sea, the Great Salt Lake, and oceanic environments with high NaCl	[235]
Metallocarboxypeptidase	*Caldithrix abyssi*	Deep-sea hydrothermal chimneys	[234]

## 8. Lipases and Esterases from Marine Extremophiles

Lipases and esterases are hydrolases that catalyze the cleavage and formation of ester bonds (EC 3.1) and belong to Carboxylic Ester Hydrolases (EC 3.1.1) (Table 6). Both types of enzymes belong to the family of serine hydrolases and share structural and functional characteristics, such as an α/β-hydrolase fold in their core structure, and have a characteristic catalytic triad (serine, aspartic/glutamic and histidine). Lipases are mainly active against water-insoluble substrates, such as triglycerides composed of long-chain fatty acids (C ≥ 8). Esterases preferentially hydrolyze “simple” esters and usually only triglycerides composed of fatty acids shorter than C 8. Based on amino acid sequences, esterases and lipases have been grouped into eight families. Enzymes in Family 1 are called true lipases and are further classified into six subfamilies. Enzymes belonging to Families 2–8 are esterases. The genome analysis of bacteria has shown putative lipases/esterases genes that are not included in any family [236,237].

**Table 6:** Lipases and esterases according the Nomenclature Committee of the International Union of Biochemistry and Molecular Biology (NC-IUBMB) [145].

Lipases	Systematic Name	E.C.	Reaction Catalysed
Carboxylesterase (other names: Esterases, serina esterases, *etc.*)	Carboxylesterase	3.1.1.1	carboxylic ester + H_2_O = alcohol + carboxylate
Triacylglycerol lipase (other names: Lipase; triglyceride lipase, *etc.*)	Triacylglycerol acylhydrolase	3.1.1.3	triacylglycerol + H_2_O = diacylglycerol + carboxylate

The biotechnological applications of lipases have a broad spectrum of use including in the food, dairy, pharmaceutical, cosmetic, agrochemical, biosurfactant, detergent and paper industries [238,239,240,241]. Biodiesel production has been one of the major sectors stimulating the search for lipases [242,243]. Biodiesel is composed of methyl-esterified fatty acids derived from transesterification of triglycerides by enzymatic action, providing a number of advantages such as a reduction of the operational process in the manufacture and separation of glycerol by-products. For the environment, the advantages are countless, such as the reduction of particles emissions, low toxicity and high biodegradability [5,244]. The genus *Pseudomonas* is the most exploited for lipases [245]. The industrial use of lipases and esterases from extremophile microorganisms has been increasing steadily in recent years [246], mainly with the use of thermostable enzymes from thermophiles and psychrophiles [247,248,249].

The *Thermococcus* genus has been isolated from black smokers (hydrothermal vents) or freshwater springs, with 1%–3% salt (NaCl) concentration, other marine hydrothermal areas and oil reservoirs. *Thermococcus sibiricus* is a hyperthermophile from a high-temperature Samotlor oil reservoir (Western Siberia). Genomic analysis revealed that *T. sibiricus* contains 15 genes that encode for lipases/esterases. Four of these putative enzymes contain signal peptides suggesting that they are extracellular enzymes [250].

A thermostable esterase gene from the aquatic hyperthermophilic *Archaeoglobus fulgidus* DSM 4304 was cloned in *E. coli*. The esterase was tested with various acyl chains of ρ-nitrophenol and the highest activity was found for ρ-nitrophenyl butyrate at 80 °C [251]. The archaeon *Pyrobaculum calidifontis* VA1, also a hyperthermophilic isolated from a hot spring in the Philippines, presented a thermostable carboxylesterase, with an optimum operation at 90 °C, and organic tolerance, supporting concentrations up to 80% [252]. Other thermostable esterases have been described in *S. solfataricus* P1. Arylesterase, the least understood among the esterases, showed high stability against denaturing agents at an optimum temperature and pH of 94 °C and 7.0, respectively [253]. Esterases have been purified and studied from the extremely *Sulfolobus acidocaldarius* [254] and *Sulfolobus shibatae* [255]. A thermostable esterase gene from *P. furiosus* has been cloned in *E. coli*, purified and studied [256].

Lipases from psychrophile microorganisms are some of the most widely used classes of enzymes in biotechnology applications, organic chemistry, detergent industry, for bioremediation purposes and in the food industry [5,12,240]. A cold-adapted lipase (M37) from *Photobacterium lipolyticum*, previously isolated from an intertidal flat of the Yellow Sea in Korea, maintains activity between 5 °C to 25 °C, and was expressed in *E. coli* at 18 °C [257]. The lipase M37 has been tested as an alternative to *C. antarctica* lipase B (Novozym435^®^—Novozymes Corporation (Bagsvaerd, Denmark), in the production of biodiesel. Novozym435 is unstable in a medium containing high concentrations of methanol, required for efficient biodiesel production [258,259]. 

*C. antarctica* was discovered to be a lipase producer in the 1980’s and it was found that two types of lipases (A and B) were being produced. Since then, innumerous applications have been described, such as the production of non-steroidal anti-inflammatory drugs (NSAIDs), biofuel and organic compounds [260,261,262]. Lipase B is commonly used for desymmetrization, ester synthesis and production of peracids [130,263]. Due to such abilities, a great effort has been made to improve the stability of lipase B through chemical modifications and directed evolution [130,261,264].

Several Antarctic isolates from Terra Nova Bay, including *Pseudoalteromonas* sp., *Psychrobacter* sp. and *Vibrio* sp. have exhibited cold adapted lipases and esterases. The enzymes of six of them work better at a 4 to 15 °C range, with pH changing from 5 to 9.0 and the NaCl concentrations between 1% and 7.0% [265]. Until now, only a few esterases from psychrophilic microorganisms have been cloned and characterized [156]. The cold-adapted esterase from *Pseudoalteromonas arctica* shows optimum activity at 25 °C, but retains more than 50% of its activity at 0 °C [266]. In addition, after the complete genome of *P. haloplanktis* was sequenced, a new cold-active lipase (Lip1) that configures a new lipolytic family was found [237]. Other psychrophilic cold-adapted lipases and esterases are summarized in Table 7.

**Table 7:** Lipases and esterases from psychrophiles.

Prokaryote	Habitat	Lipase/Esterase	Reference
*Desulfotalea psychrophila (sulfate-reducing bacteria)*	Marine sediments that are permanently cold	Esterase	[267]
*Pseudoalteromonas haloplanktis*	Marine Antarctic	Lipase	[237]
*Psychrobacter* sp. wp37	Deep-sea sediments	Lipase	[268]
*Colwellia psychrerythraea* 34H (recombinant in *E. coli*)	Cold marine environments including Arctic and Antarctic sea ice	Lipase	[269]

One halotolerant strain identified as *Salinivibrio* sp. strain SA-2 was isolated from a hypersaline brackish water with 14% salinity (Garmsar-Iran) and presented a thermostable lipase, retaining 90% of its activity at 80 °C in a pH range of 7.5–8 for 30 min, tolerating a range of 0 to 3.0 M NaCl concentrations [270]. The Haloarcula marismortui is an extreme halophilic archaeon isolated from the Dead Sea, a hypersaline lake, producing different enzymes such as alkaline phosphatase, lipases, alcohol dehydrogenases and other bio-products [271]. An esterase from *H. marismortui* was expressed in *E. coli* and has been characterized as a salt-dependent esterase, with maximum activity at 3.0 M of KCl and no activity in the absence of this salt [272]. A lipase was isolated from *Natronococcus* sp., an extremely halophilic archaeon [273]. According to Ferrer *et al.* [11], five esterase genes were found in the Urania deep-sea hypersaline anoxic basins (Eastern Mediterranean), through a screening in the metagenome expression library. After expression in *E. coli* at least two were able to function in an extreme condition, with high-pressure and saline affinity, and one possesses an adaptive structure, showing halotolerance and conferring a wide range of catalytic activities. These esterases may possibly take part in the production of intermediate pharmaceuticals, synthesizing optically pure biological active substances.

## 9. Conclusions and Future Perspectives

The study of marine extremophiles has been significantly reinforced using the modern techniques of bioengineering and molecular biology. Such interactions have favored a better understanding of these microorganisms and their biotechnological applications. The evolutionary adaptation to extreme conditions has facilitated the selection of a more tolerant metabolism, of enzymes and other products with special features not found in any other prokaryotes. Good examples are hydrolases with the ability to operate in temperatures as low as those of the microorganisms isolated from Antarctica to those active at high temperatures such as the hyperthermophilic microorganisms isolated from thermal fractures; enzymes that act in high concentrations of salts found in anoxic basin environments, or high pressure, are found in several polyextremophiles. These properties make these unique microorganisms a source of genes that may be mapped by bio-prospecting metagenomic studies and used for cloning and expression in other organisms to obtain enzymes with properties suitable for industrial bioprocesses. However, although the studies related to the marine extremophiles cited here have increased greatly over the past decade, there are still other extremozymes in promising habitats such as deep sea floors, and also in the Red Sea that have so far attracted few studies and are open for exploration. Marine extremophiles are a group of prokaryotic organisms that are being intensively studied worldwide. Although major advances have been made recently, our knowledge of the physiology, metabolism, enzymology and genetics of this fascinating group of microorganisms is still limited. Therefore, an important growth is expected to take place in this sector, pursuing a better understanding of the application of these hydrolases from marine extremophiles.

## References

[1] Bull A.T., Ward A.C., Goodfellow M. (2000). Search and discovery strategies for biotechnology: The paradigm shift. Microbiol. Mol. Biol. Rev..

[2] Nath I.V.A., Bharathi P.A.L. (2011). Diversity in transcripts and translational pattern of stress proteins in marine extremophiles. Extremophiles.

[3] BCC Research (2014). Global Markets for Enzymes in Industrial Applications.

[4] Díaz-Tenaa E., Rodríguez-Ezquerroa A., Marcaidea L.N.L.L., Bustinduyb L.G., Sáenzb A.E. (2013). Use of Extremophiles Microorganisms for Metal Removal. Procedia Eng..

[5] Vermelho A.B., Noronha E.F., Filho E.X., Ferrara M.A., Bon E.P.S., Rosenberg E., DeeLong E.F., Lory S., Stackebrandt E., Thompson F. (2013). Diversity and biotechnological applications of prokaryotic enzymes. The Prokaryotes.

[6] Chandrasekaran M., Kumar S.R., Werner H., Roken S. (2010). Marine microbial enzymes. Biotechnology.

[7] Fulzele R., Desa E., Yadav A., Shouche Y., Bhadekar R. (2011). Characterization of novel extracellular protease produced by marine bacterial isolate from the Indian Ocean. Braz. J. Microbiol..

[8] Samuel P., Raja A., Prabakaran P. (2012). Investigation and application of marine derived microbial enzymes: Status and prospects. Int. J. Ocean. Mar. Ecol. Syst..

[9] Russo R., Giordano D., Riccio A., di Prisco G., Verde C. (2010). Cold-adapted bacteria and the globin case study in the Antarctic bacterium *Pseudoalteromonas haloplanktis* TAC125. Mar. Genomics.

[10] Trincone A. (2010). Potential biocatalysts originating from sea environments. J. Mol. Catal. B-Enzym..

[11] Ferrer M., Golyshina O.V., Chernikova T.N., Khachane A.N., Martins Dos Santos V.A., Yakimov M.M., Timmis K.N., Golyshin P.N. (2005). Microbial enzymes mined from the Urania deep-sea hypersaline anoxic basin. Chem. Biol..

[12] Joseph B., Ramteke P.W., Thomas G. (2008). Cold active microbial lipases: Some hot issues and recent developments. Biotechnol. Adv..

[13] Hicks P.M., Rinker K.D., Baker J.R., Kelly R.M. (1998). Homomultimeric protease in the hyperthermophilic bacterium *Thermotoga maritima* has structural and amino acid sequence homology to bacteriocins in mesophilic bacteria. FEBS Lett..

[14] Valdes-Stauber N., Scherer S. (1996). Nucleotide sequence and taxonomical distribution of the bacteriocin gene lin cloned from *Brevibacterium linens* M18. Appl. Environ. Microbiol..

[15] Zhan D., Sun J., Feng Y., Han W. (2014). Theoretical study on the allosteric regulation of an oligomeric protease from *Pyrococcus horikoshii* by Cl-Ion. Molecules.

[16] Kim J., Dordick J.S. (1997). Unusual salt and solvent dependence of a protease from an extreme halophile. Biotechnol. Bioeng..

[17] Horikoshi K., Bull A.T., Horikoshi K., Antranikaian G., Bull A.T., Robb F.T., Stetter K.O. (2011). Prologue: Definition, categories, distribution, origin and evolution, pioneering studies, and emerging fields of extremophiles. Extremophiles Handbook.

[18] Stan-Latter H., Stan-Latter H., Fendrihan S. (2012). Physico-chemical boundaries of life. Adaption of Microbial Life to Environmental Extremes.

[19] Horikoshi K., Antranikaian G., Bull A.T., Robb F.T., Stetter K.O. (2011). Extremophiles Handbook.

[20] Seckbach J., Oren A., Stan-Latter H. (2013). Polyextremophiles: Life Under Multiple Forms of Stress.

[21] Arora R., Bell E.M., Bell E.M. (2012). Biotechnological applications of extremophiles: Promise and prospects. Life at Extremes: Environments, Organisms and Strategies for Survival.

[22] Gabani P., Singh O.V. (2013). Radiation-resistant extremophiles and their potential in biotechnology and therapeutics. Appl. Microbiol. Biotechnol..

[23] Karan R., Capes M.D., Dassarma S. (2012). Function and biotechnology of extremophilic enzymes in low water activity. Aquat. Biosyst..

[24] Morozkina E.V., Slutskaia E.S., Fedorova T.V., Tugai T.I., Golubeva L.I., Koroleva O.V. (2010). Extremophilic microorganisms: Biochemical adaptation and biotechnological application (review). Prikl. Biokhim. Mikrobiol..

[25] Singh O.V. (2012). Extremophiles: Sustainable Resources and Biotechnological Implications.

[26] Gelfand D.H., Stoffel S., Lawyer F.C., Saiki R.K. (1989). Purified Thermostable Enzyme.

[27] Rampelotto P.H. (2013). Biotechnological applications of extremophiles. Curr. Biotechnol..

[28] Woese C.R., Kandler O., Wheelis M.L. (1990). Towards a natural system of organisms: Proposal for the domains Archaea, Bacteria, and Eucarya. Proc. Natl. Acad. Sci. USA.

[29] Lang J.M., Darling A.E., Eisen J.A. (2013). Phylogeny of bacterial and archaeal genomes using conserved genes: Supertrees and supermatrices. PLoS ONE.

[30] Dereeper A., Guignon V., Blanc G., Audic S., Buffet S., Chevenet F., Dufayard J.F., Guindon S., Lefort V., Lescot M. (2008). Phylogeny.fr: Robust phylogenetic analysis for the non-specialist. Nucleic Acids Res..

[31] Pikuta E.V., Hoover R.B., Tang J. (2007). Microbial extremophiles at the limits of life. Crit. Rev. Microbiol..

[32] Rothschild L.J., Mancinelli R.L. (2001). Life in extreme environments. Nature.

[33] Stetter K.O., Horikoshi K., Antranikaian G., Bull A.T., Robb F.T., Stetter K.O. (2011). History of discovery of hyperthermophiles. Extremophiles Handbook.

[34] Canganella F., Wiegel J. (2014). Anaerobic thermophiles. Life.

[35] Takai K., Nakamura K., Toki T., Tsunogai U., Miyazaki M., Miyazaki J., Hirayama H., Nakagawa S., Nunoura T., Horikoshi K. (2008). Cell proliferation at 122 degrees C and isotopically heavy CH4 production by a hyperthermophilic methanogen under high-pressure cultivation. Proc. Natl. Acad. Sci. USA.

[36] Fushida S., Mizuno Y., Masuda H., Toki T., Makita H. (2014). Concentrations and distributions of amino acids in black and white smoker fluids at temperatures over 200 °C. Org. Geochem..

[37] White R.H. (1984). Hydrolytic stability of biomolecules at high temperatures and its implication for life at 250 degrees C. Nature.

[38] Colletier J.P., Aleksandrov A., Coquelle N., Mraihi S., Mendoza-Barbera E., Field M., Madern D. (2012). Sampling the conformational energy landscape of a hyperthermophilic protein by engineering key substitutions. Mol. Biol. Evol..

[39] Tehei M., Madern D., Franzetti B., Zaccai G. (2005). Neutron scattering reveals the dynamic basis of protein adaptation to extreme temperature. J. Biol. Chem..

[40] Aung H.L., Samaranayaka C.U., Enright R., Beggs K.T., Monk B.C. (2015). Characterisation of the DNA gyrase from the thermophilic eubacterium *Thermus thermophilus*. Protein. Expr. Purif..

[41] Hidalgo A., Berenguer J. (2013). Biotechnological applications of *Thermus thermophilus* as host. Curr. Biotechnol..

[42] Sakaff M.K.L.M., Rahman A.Y.A., Saito J.A., Hou S.B., Alam M. (2012). Complete genome sequence of the thermophilic bacterium *Geobacillus thermoleovorans* CCB_US3_UF5. J. Bacteriol..

[43] Hurst L.D., Merchant A.R. (2001). High guanine-cytosine content is not an adaptation to high temperature: A comparative analysis amongst prokaryotes. Proc. Biol. Sci..

[44] Reed C.J., Lewis H., Trejo E., Winston V., Evilia C. (2013). Protein adaptations in archaeal extremophiles. Archaea.

[45] Koga Y. (2012). Thermal adaptation of the archaeal and bacterial lipid membranes. Archaea.

[46] Liszka M.J., Clark M.E., Schneider E., Clark D.S. (2012). Nature *versus* nurture: Developing enzymes that function under extreme conditions. Annu. Rev. Chem. Biomol. Eng..

[47] Klippel B., Antranikian G., Horikoshi K., Antranikaian G., Bull A.T., Robb F.T., Stetter K.O. (2011). Lignocellulose converting enzymes from thermophiles. Extremophiles Handbook.

[48] Turner P., Mamo G., Karlsson E.N. (2007). Potential and utilization of thermophiles and thermostable enzymes in biorefining. Microb. Cell. Fact..

[49] Yeoman C.J., Han Y., Dodd D., Schroeder C.M., Mackie R.I., Cann I.K. (2010). Thermostable enzymes as biocatalysts in the biofuel industry. Adv. Appl. Microbiol..

[50] Junge K., Christner B., Staley J.T., Horikoshi K., Antranikaian G., Bull A.T., Robb F.T., Stetter K.O. (2011). Diversity of psychrophilic bacteria from sea ice—and glacial ice communities. Extremophiles Handbook.

[51] Cavicchioli R. (2006). Cold-adapted archaea. Nat. Rev. Microbiol..

[52] Margesin R., Miteva V. (2011). Diversity and ecology of psychrophilic microorganisms. Res. Microbiol..

[53] Bakermans C., Bergholz P.W., Rodrigues D., Vishnevetskaya T.A., Ayala-del-Río H.L., Tiedje J., Miller R.V., Whyte L.G. (2011). Genomic and expression analyses of cold-adapted microorganisms. Polar Microbiology: Life in a Deep Freeze.

[54] Mykytczuk N.C., Foote S.J., Omelon C.R., Southam G., Greer C.W., Whyte L.G. (2013). Bacterial growth at −15 °C; molecular insights from the permafrost bacterium *Planococcus halocryophilus* Or1. ISME J..

[55] Kurosawa N., Sato S., Kawarabayasi Y., Imura S., Naganuma T. (2010). Archaeal and bacterial community structures in the anoxic sediment of Antarctic meromictic lake Nurume-Ike. Polar Sci..

[56] Siddiqui K.S., Williams T.J., Wilkins D., Yau S., Allen M.A., Brown M.V., Lauro F.M., Cavicchioli R. (2013). Psychrophiles. Annu. Rev. Earth Planet. Sci..

[57] Jones P.G., Inouye M. (1994). The cold-shock response--a hot topic. Mol. Microbiol..

[58] Lim J., Thomas T., Cavicchioli R. (2000). Low temperature regulated DEAD-box RNA helicase from the Antarctic archaeon, *Methanococcoides burtonii*. J. Mol. Biol..

[59] Noon K.R., Guymon R., Crain P.F., McCloskey J.A., Thomm M., Lim J., Cavicchioli R. (2003). Influence of temperature on tRNA modification in Archaea: *Methanococcoides burtonii* (optimum growth temperature [T_opt_], 23 °C) and *Stetteria hydrogenophila* (T_opt_, 95 °C). J. Bacteriol..

[60] Casanueva A., Tuffin M., Cary C., Cowan D.A. (2010). Molecular adaptations to psychrophily: The impact of “omic” technologies. Trends Microbiol..

[61] De Maayer P., Anderson D., Cary C., Cowan D.A. (2014). Some like it cold: Understanding the survival strategies of psychrophiles. EMBO Rep..

[62] Lorv J.S., Rose D.R., Glick B.R. (2014). Bacterial ice crystal controlling proteins. Scientifica.

[63] Madigan M.T., Martinko J.M., Dunlap P.V., Clarck D.P. (2014). Brock Biology of Microorganisms.

[64] Deming J.W., Schaechter M. (2009). Extremophiles: Cold environments. The Desk Encyclopedia of Microbiology.

[65] Joshi S., Satyanarayana T. (2013). Biotechnology of cold-active proteases. Biology.

[66] Siddiqui K.S., Cavicchioli R. (2006). Cold-adapted enzymes. Annu. Rev. Biochem..

[67] Fendriham S., Negoiţă T.G., Stan-Latter H., Fendrihan S. (2012). Psychrophilic microorganisms as important source for biotechnological processes. Adaption of Microbial Life to Environmental Extremes.

[68] Huston A.L., Margesin R., Schinner F., Marx J.-C., Gerday C. (2008). Biotechnological aspects of cold adapted enzymes. Psychrophiles: From Biodiversity to Biotechnology.

[69] Feller G. (2013). Psychrophilic enzymes: From folding to function and biotechnology. Scientifica.

[70] Jackson R.B., Carpenter S.R., Dahm C.N., McKnight D.M., Naiman R.J., Postel S.L., Running S.W. (2001). Water in a changing world. Ecol. Appl..

[71] Siglioccolo A., Paiardini A., Piscitelli M., Pascarella S. (2011). Structural adaptation of extreme halophilic proteins through decrease of conserved hydrophobic contact surface. BMC Struct. Biol..

[72] Javor B. (1989). Deep sea hypersaline basins. Hypersaline Environments.

[73] Van der Wielen P.W., Bolhuis H., Borin S., Daffonchio D., Corselli C., Giuliano L., D’Auria G., de Lange G.J., Huebner A., Varnavas S.P. (2005). The enigma of prokaryotic life in deep hypersaline anoxic basins. Science.

[74] Yakimov M., La Cono V., Ferrer M., Golyshin P., Giuliano L., Nelson K.E. (2014). Metagenomics of deep hypersaline anoxic basins. Encyclopedia of Metagenomics.

[75] Mapelli F., Borin S., Daffonchio D., Stan-Lotter H., Fendrihan S. (2012). Microbial diversity in deep hypersaline anoxic basins. Adaption of Microbial Life to Environmental Extremes.

[76] Hallsworth J.E., Yakimov M.M., Golyshin P.N., Gillion J.L., D’Auria G., de Lima Alves F., La Cono V., Genovese M., McKew B.A., Hayes S.L. (2007). Limits of life in MgCl_2_-containing environments: Chaotropicity defines the window. Environ. Microbiol..

[77] McGenity T.J., Oren A., Bell E.M. (2012). Hypersaline environments. Life at Extremes: Environments, Organisms and Strategies for Survival.

[78] Antunes A., Ngugi D.K., Stingl U. (2011). Microbiology of the Red Sea (and other) deep-sea anoxic brine lakes. Environ. Microbiol. Rep..

[79] Oren A. (2008). Microbial life at high salt concentrations: Phylogenetic and metabolic diversity. Saline Syst..

[80] Moreno M.L., Pérez D., García M.T., Mellado E. (2013). Halophilic bacteria as a source of novel hydrolytic enzymes. Life.

[81] Ginzburg M., Sachs L., Ginzburg B.Z. (1970). Ion metabolism in a *Halobacterium*. I. Influence of age of culture on intracellular concentrations. J. Gen. Physiol..

[82] Lanyi J.K., Silverman M.P. (1972). The state of binding of intracellular K + in *Halobacterium cutirubrum*. Can. J. Microbiol..

[83] Roberts M.F. (2005). Organic compatible solutes of halotolerant and halophilic microorganisms. Saline Syst..

[84] Tadeo X., Lopez-Mendez B., Trigueros T., Lain A., Castano D., Millet O. (2009). Structural basis for the aminoacid composition of proteins from halophilic archaea. PLoS Biol..

[85] DasSarma S., DasSarma P. (2012). Halophiles. Els.

[86] Tokunaga H., Arakawa T., Tokunaga M. (2008). Engineering of halophilic enzymes: Two acidic amino acid residues at the carboxy-terminal region confer halophilic characteristics to *Halomonas* and *Pseudomonas* nucleoside diphosphate kinases. Protein Sci..

[87] Delgado-Garcia M., Valdivia-Urdiales B., Aguilar-Gonzalez C.N., Contreras-Esquivel J.C., Rodriguez-Herrera R. (2012). Halophilic hydrolases as a new tool for the biotechnological industries. J. Sci. Food Agric..

[88] Eichler J. (2003). Facing extremes: Archaeal surface-layer (glyco)proteins. Microbiology.

[89] Enache M., Popescu G., Itoh T., Kamekura M., Stan-Latter H., Fendrihan S. (2012). Halophilic microorganisms from man-made and natural hypersaline environments: Physiology, ecology, and biotechnological potential. Adaption of Microbial Life to Environmental Extremes.

[90] Le Borgne S., Paniagua D., Vazquez-Duhalt R. (2008). Biodegradation of organic pollutants by halophilic bacteria and archaea. J. Mol. Microbiol. Biotechnol..

[91] Abe F., Horikoshi K. (2001). The biotechnological potential of piezophiles. Trends Biotechnol..

[92] Bartlett D.H., Bidle K.A., Seckbach J. (1999). Membrane-based adaptations of deep-sea piezophiles. Enigmatic Microorganisms and Life in Extreme Environments.

[93] Mota M.J., Lopes R.P., Delgadillo I., Saraiva J.A. (2013). Microorganisms under high pressure-adaptation, growth and biotechnological potential. Biotechnol. Adv..

[94] Kato C., Horikoshi K., Antranikaian G., Bull A.T., Robb F.T., Stetter K.O. (2011). High pressure and prokaryotes. Extremophiles Handbook.

[95] Oger P., Cario A. (2014). [The high pressure life of piezophiles]. Biol. Aujourdhui.

[96] Bartlett D.H. (2002). Pressure effects on *in vivo* microbial processes. Biochim. Biophys. Acta.

[97] Lauro F.M., Bartlett D.H. (2008). Prokaryotic lifestyles in deep sea habitats. Extremophiles.

[98] Simonato F., Campanaro S., Lauro F.M., Vezzi A., D’Angelo M., Vitulo N., Valle G., Bartlett D.H. (2006). Piezophilic adaptation: A genomic point of view. J. Biotechnol..

[99] Lauro F.M., Chastain R.A., Blankenship L.E., Yayanos A.A., Bartlett D.H. (2007). The unique 16S rRNA genes of piezophiles reflect both phylogeny and adaptation. Appl. Environ. Microbiol..

[100] Capece M.C., Clark E., Saleh J.K., Halford D., Heinl N., Hoskins S., Rothschild L.J., Seckbach J., Oren A., Stan-Latter H. (2013). Polyextremophiles and the constraints for terrestrial habitability. Polyextremophiles: Life under Multiple Forms of Stress.

[101] Yumoto I., Hirota K., Nodasaka Y., Yokota Y., Hoshino T., Nakajima K. (2004). *Alkalibacterium psychrotolerans* sp. nov., a psychrotolerant obligate alkaliphile that reduces an indigo dye. Int. J. Syst. Evol. Microbiol..

[102] Padan E., Bibi E., Ito M., Krulwich T.A. (2005). Alkaline pH homeostasis in bacteria: New insights. Biochim. Biophys. Acta.

[103] Baker-Austin C., Dopson M. (2007). Life in acid: pH homeostasis in acidophiles. Trends Microbiol..

[104] Wiegel J., Horikoshi K., Antranikaian G., Bull A.T., Robb F.T., Stetter K.O. (2011). Anaerobic alkaliphiles and alkaliphilic poly-extremophiles. Extremophiles Handbook.

[105] Fukuchi S., Yoshimune K., Wakayama M., Moriguchi M., Nishikawa K. (2003). Unique amino acid composition of proteins in halophilic bacteria. J. Mol. Biol..

[106] Zaccai G., Gargaud M., López-García P., Martin H. (2011). Molecular adaptations to life in high salt: Lessons from *Haloarcula marismortui*. Origins and Evolution of Life: An Astrobiological Perspective.

[107] Gonzalez J.M., Sheckells D., Viebahn M., Krupatkina D., Borges K.M., Robb F.T. (1999). *Thermococcus waiotapuensis* sp. nov., an extremely thermophilic archaeon isolated from a freshwater hot spring. Arch. Microbiol..

[108] Vanlint D., Michiels C.W., Aertsen A., Horikoshi K., Antranikaian G., Bull A.T., Robb F.T., Stetter K.O. (2011). Piezophysiology of the model bacterium *Escherichia coli*. Extremophiles Handbook.

[109] Kato C., Li L., Nogi Y., Nakamura Y., Tamaoka J., Horikoshi K. (1998). Extremely barophilic bacteria isolated from the Mariana Trench, Challenger Deep, at a depth of 11,000 meters. Appl. Environ. Microbiol..

[110] Blum J.S., Bindi A.B., Buzzelli J., Stolz J.F., Oremland R.S. (1998). *Bacillus arsenicoselenatis*, sp. nov., and *Bacillus selenitireducens*, sp. nov.: Two haloalkaliphiles from Mono Lake, California that respire oxyanions of selenium and arsenic. Arch. Microbiol..

[111] Mesbah N.M., Hedrick D.B., Peacock A.D., Rohde M., Wiegel J. (2007). *Natranaerobius thermophilus* gen. nov., sp. nov., a halophilic, alkalithermophilic bacterium from soda lakes of the Wadi An Natrun, Egypt, and proposal of *Natranaerobiaceae* fam. nov. and *Natranaerobiales* ord. nov. Int. J. Syst. Evol. Microbiol..

[112] Mesbah N.M., Wiegel J., Ventosa A., Oren A., Ma Y. (2011). Halophiles exposed concomitantly to multiple stressors: Adaptive mechanisms of halophilic alkalithermophiles. Halophiles and Hypersaline Environments.

[113] Abe F., Kato C., Horikoshi K. (1999). Pressure-regulated metabolism in microorganisms. Trends Microbiol..

[114] Kumar V., Satyanarayana T. (2012). Thermo-alkali-stable xylanase of a novel polyextremophilic *Bacillus halodurans* TSEV1 and its application in biobleaching. Int. Biodeter. Biodegr..

[115] Kumar V., Satyanarayana T. (2015). Generation of xylooligosaccharides from microwave irradiated agroresidues using recombinant thermo-alkali-stable endoxylanase of the polyextremophilic bacterium *Bacillus halodurans* expressed in *Pichia pastoris*. Bioresour. Technol..

[116] Vijayalaxmi S., Prakash P., Jayalakshmi S.K., Mulimani V.H., Sreeramulu K. (2013). Production of extremely alkaliphilic, halotolerent, detergent, and thermostable mannanase by the free and immobilized cells of *Bacillus halodurans* PPKS-2. Purification and characterization. Appl. Biochem. Biotechnol..

[117] Karan R., Capes M.D., DasSarma P., DasSarma S. (2013). Cloning, overexpression, purification, and characterization of a polyextremophilic beta-galactosidase from the Antarctic haloarchaeon *Halorubrum lacusprofundi*. BMC Biotechnol..

[118] Bommarius A.S., Blum J.K., Abrahamson M.J. (2011). Status of protein engineering for biocatalysts: How to design an industrially useful biocatalyst. Curr. Opin. Chem. Biol..

[119] Kumar A., Singh S. (2013). Directed evolution: Tailoring biocatalysts for industrial applications. Crit. Rev. Biotechnol..

[120] Chen Z., Wilmanns M., Zeng A.P. (2010). Structural synthetic biotechnology: From molecular structure to predictable design for industrial strain development. Trends Biotechnol..

[121] Otero J.M., Nielsen J. (2010). Industrial systems biology. Biotechnol. Bioeng..

[122] Venkatesh R., Sundaram P.V. (1998). Upward shift of thermotolerance of cold water fish and mammalian trypsins upon chemical modification. Ann. N. Y. Acad. Sci..

[123] Venkatesh R., Sundaram P.V. (1998). Modulation of stability properties of bovine trypsin after *in vitro* structural changes with a variety of chemical modifiers. Protein Eng..

[124] Arnold F.H. (2001). Combinatorial and computational challenges for biocatalyst design. Nature.

[125] Bornscheuer U.T., Huisman G.W., Kazlauskas R.J., Lutz S., Moore J.C., Robins K. (2012). Engineering the third wave of biocatalysis. Nature.

[126] Reetz M.T. (2011). Laboratory evolution of stereoselective enzymes: A prolific source of catalysts for asymmetric reactions. Angew. Chem. Int. Ed..

[127] Kourist R., Bornscheuer U.T. (2011). Biocatalytic synthesis of optically active tertiary alcohols. Appl. Microbiol. Biotechnol..

[128] Kourist R., Dominguez de Maria P., Miyamoto K. (2011). Biocatalytic strategies for the asymmetric synthesis of profens—Recent trends and developments. Green Chem..

[129] Eijsink V.G., Bjork A., Gaseidnes S., Sirevag R., Synstad B., van den Burg B., Vriend G. (2004). Rational engineering of enzyme stability. J. Biotechnol..

[130] Siddiqui K.S., Cavicchioli R. (2005). Improved thermal stability and activity in the cold-adapted lipase B from *Candida antarctica* following chemical modification with oxidized polysaccharides. Extremophiles.

[131] Shu Z.-Y., Jiang H., Lin R.-F., Jiang Y.-M., Lin L., Huang J.-Z. (2010). Technical methods to improve yield, activity and stability in the development of microbial lipases. J. Mol. Catal. B: Enzym..

[132] Siddiqui K.S., Poljak A., Guilhaus M., De Francisci D., Curmi P.M., Feller G., D’Amico S., Gerday C., Uversky V.N., Cavicchioli R. (2006). Role of lysine *versus* arginine in enzyme cold-adaptation: Modifying lysine to homo-arginine stabilizes the cold-adapted alpha-amylase from *Pseudoalteramonas haloplanktis*. Proteins.

[133] Ferrer M., Martinez-Abarca F., Golyshin P.N. (2005). Mining genomes and “metagenomes” for novel catalysts. Curr. Opin. Biotechnol..

[134] Handelsman J. (2004). Metagenomics: Application of genomics to uncultured microorganisms. Microbiol. Mol. Biol. Rev..

[135] Lopez-Lopez O., Cerdan M.E., Gonzalez-Siso M.I. (2014). New extremophilic lipases and esterases from metagenomics. Curr. Protein Pept. Sci..

[136] Kennedy J., Flemer B., Jackson S.A., Lejon D.P., Morrissey J.P., O’Gara F., Dobson A.D. (2010). Marine metagenomics: New tools for the study and exploitation of marine microbial metabolism. Mar. Drugs.

[137] Kotera M., Moriya Y., Tokimatsu T., Kanehisa M., Goto S., Nelson K.E. (2014). KEGG and GenomeNet, new developments, metagenomic analysis. Encyclopedia of Metagenomics.

[138] Blaser H.U. (2003). Enantioselective catalysis in fine chemicals production. Chem. Commun..

[139] Miyake R., Kawamoto J., Wei Y.L., Kitagawa M., Kato I., Kurihara T., Esaki N. (2007). Construction of a low-temperature protein expression system using a cold-adapted bacterium, *Shewanella* sp. strain Ac10, as the host. Appl. Environ. Microbiol..

[140] Tajima T., Fuki K., Kataoka N., Kudou D., Nakashimada Y., Kato J. (2013). Construction of a simple biocatalyst using psychrophilic bacterial cells and its application for efficient 3-hydroxypropionaldehyde production from glycerol. AMB Express.

[141] Ferrer M., Chernikova T.N., Yakimov M.M., Golyshin P.N., Timmis K.N. (2003). Chaperonins govern growth of *Escherichia coli* at low temperatures. Nat. Biotechnol..

[142] Adrio J.L., Demain A.L. (2014). Microbial enzymes: Tools for biotechnological processes. Biomolecules.

[143] Binod P., Palkhiwala P., Gaikaiwari R., Nampoothiri K.M., Duggal A., Dey K., Pandey A. (2013). Industrial enzymes—Present status and future perspectives for India. J. Sci. Ind. Res. India.

[144] Kumar L., Awasthi G., Singh B. (2011). Extremophiles: A novel source of industrially important enzymes. Biotechnol. Appl. Biochem..

[145] NC-IUBMB. The Enzyme List Class 3—Hydrolases.

[146] Zeeman S.C., Kossmann J., Smith A.M. (2010). Starch: Its metabolism, evolution, and biotechnological modification in plants. Annu. Rev. Plant Biol..

[147] Castro A.M., Ribeiro B.D., Vermelho A.B., Couri S. (2013). Methods for detection of amylolytic activities. Methods to Determine Enzymatic Activity.

[148] Prakash O., Jaiswal N. (2010). alpha-Amylase: An ideal representative of thermostable enzymes. Appl. Biochem. Biotechnol..

[149] Feller G., Gerday C. (2003). Psychrophilic enzymes: Hot topics in cold adaptation. Nat. Rev. Microbiol..

[150] Kuddus M., Roohi, Arif J.M., Ramteke P.W. (2011). An overview of cold-active microbial α-amylase: Adaptation strategies and biotechnological potentials. Biotechnology.

[151] Niehaus F., Bertoldo C., Kahler M., Antranikian G. (1999). Extremophiles as a source of novel enzymes for industrial application. Appl. Microbiol. Biotechnol..

[152] Sharma A., Satyanarayana T. (2013). Microbial acid-stable α-amylases: Characteristics, genetic engineering and applications. Process Biochem..

[153] Brown I., Dafforn T.R., Fryer P.J., Cox P.W. (2013). Kinetic study of the thermal denaturation of a hyperthermostable extracellular alpha-amylase from *Pyrococcus furiosus*. Biochim. Biophys. Acta.

[154] Duffner F., Bertoldo C., Andersen J.T., Wagner K., Antranikian G. (2000). A new thermoactive pullulanase from *Desulfurococcus mucosus*: Cloning, sequencing, purification, and characterization of the recombinant enzyme after expression in *Bacillus subtilis*. J. Bacteriol..

[155] Gomes I., Gomes J., Steiner W. (2003). Highly thermostable amylase and pullulanase of the extreme thermophilic eubacterium *Rhodothermus marinus:* Production and partial characterization. Bioresour. Technol..

[156] Trincone A. (2011). Marine biocatalysts: Enzymatic features and applications. Mar. Drugs.

[157] Gurumurthy D.M., Neelagund S.E. (2012). Molecular characterization of industrially viable extreme thermostable novel alpha-amylase of *Geobacillus* sp. Iso5 isolated from geothermal spring. J. Pure Appl. Microbiol..

[158] Anto H., Trivedi U.B., Patel K.C. (2006). Glucoamylase production by solid-state fermentation using rice flake manufacturing waste products as substrate. Bioresour. Technol..

[159] Sun H., Zhao P., Ge X., Xia Y., Hao Z., Liu J., Peng M. (2010). Recent advances in microbial raw starch degrading enzymes. Appl. Biochem. Biotechnol..

[160] Kyaw N., de Mesquita R.F., Kameda E., Neto J.C., Langone M.A., Coelho M.A. (2010). Characterization of commercial amylases for the removal of filter cake on petroleum wells. Appl. Biochem. Biotechnol..

[161] Sivaramakrishnan S., Gangadharan D., Nampoothiri K.M., Pandey A. (2006). Amylases from microbial sources—An overview on recent developments. Food Technol. Biotechnol..

[162] Callen W., Richardson T., Frey G., Miller C., Kazaoka M., Mathur E., Short J. (2012). Amylases and Methods for Use in Starch Processing.

[163] Nedwin G.E., Sharma V., Shetty J.K. (2013). Alpha-Amylase Blend for Starch Processing and Method of Use Thereof.

[164] Hii S.L., Tan J.S., Ling T.C., Ariff A.B. (2012). Pullulanase: Role in starch hydrolysis and potential industrial applications. Enzyme Res..

[165] Fernandes P. (2014). Marine enzymes and food industry: Insight on existing and potential interactions. Front. Mar. Sci..

[166] Lévêque E., Janeček Š., Haye B., Belarbi A. (2000). Thermophilic archaeal amylolytic enzymes. Enzym. Microb. Tech..

[167] Vieille C., Zeikus G.J. (2001). Hyperthermophilic enzymes: Sources, uses, and molecular mechanisms for thermostability. Microbiol. Mol. Biol. Rev..

[168] Li X., Li D., Park K.H. (2013). An extremely thermostable amylopullulanase from *Staphylothermus marinus* displays both pullulan- and cyclodextrin-degrading activities. Appl. Microbiol. Biotechnol..

[169] Brown S.H., Kelly R.M. (1993). Characterization of amylolytic enzymes, having both alpha-1,4 and alpha-1,6 hydrolytic activity, from the thermophilic Archaea *Pyrococcus furiosus* and *Thermococcus litoralis*. Appl. Environ. Microbiol..

[170] Feller G., Le Bussy O., Gerday C. (1998). Expression of psychrophilic genes in mesophilic hosts: Assessment of the folding state of a recombinant alpha-amylase. Appl. Environ. Microbiol..

[171] Srimathi S., Jayaraman G., Feller G., Danielsson B., Narayanan P.R. (2007). Intrinsic halotolerance of the psychrophilic alpha-amylase from *Pseudoalteromonas haloplanktis*. Extremophiles.

[172] Qin Y., Huang Z., Liu Z. (2014). A novel cold-active and salt-tolerant alpha-amylase from marine bacterium *Zunongwangia profunda:* Molecular cloning, heterologous expression and biochemical characterization. Extremophiles.

[173] Pancha I., Jain D., Shrivastav A., Mishra S.K., Shethia B., Mishra S., V P.M., Jha B. (2010). A thermoactive alpha-amylase from a *Bacillus* sp. isolated from CSMCRI salt farm. Int. J. Biol. Macromol..

[174] Uzyol K.S., Sarıyar-Akbulut B., Denizci A.A., Kazan D. (2012). Thermostable α-amylase from moderately halophilic *Halomonas* sp. AAD21. Turk. J. Biol..

[175] Fukushima T., Mizuki T., Echigo A., Inoue A., Usami R. (2005). Organic solvent tolerance of halophilic alpha-amylase from a Haloarchaeon, *Haloarcula* sp. strain S-1. Extremophiles.

[176] Laderman K.A., Asada K., Uemori T., Mukai H., Taguchi Y., Kato I., Anfinsen C.B. (1993). Alpha-amylase from the hyperthermophilic archaebacterium *Pyrococcus furiosus*. Cloning and sequencing of the gene and expression in *Escherichia coli*. J. Biol. Chem..

[177] Bertoldo C., Duffner F., Jorgensen P.L., Antranikian G. (1999). Pullulanase type I from *Fervidobacterium pennavorans* Ven5: Cloning, sequencing, and expression of the gene and biochemical characterization of the recombinant enzyme. Appl. Environ. Microbiol..

[178] Bhattacharya A., Pletschke B.I. (2014). Review of the enzymatic machinery of *Halothermothrix orenii* with special reference to industrial applications. Enzyme Microb. Technol..

[179] Perez-Pomares F., Bautista V., Ferrer J., Pire C., Marhuenda-Egea F.C., Bonete M.J. (2003). Alpha-amylase activity from the halophilic archaeon *Haloferax mediterranei*. Extremophiles.

[180] Tomme P., Warren R.A., Gilkes N.R., Stafford R. (1995). Cellulose hydrolysis by bacteria and fungi. Advances in Microbial Physiology.

[181] Wilson D.B., Himmel M.E. (2008). Aerobic microbial cellulase systems. Biomass Recalcitrance: Deconstructing the Plant Cell Wall for Bioenergy.

[182] Collins T., Gerday C., Feller G. (2005). Xylanases, xylanase families and extremophilic xylanases. FEMS Microbiol. Rev..

[183] Ratanakhanokchai K., Waeonukul R., Pason P., Pason C., Kyu K.L., SakkaK, Kosugi A., Mori Y., Matovic M.D. (2013). *Paenibacillus curdlanolyticus* strain B-6 multienzyme complex: A novel system for biomass utilization. Biomass Now—Cultivation and Utilization.

[184] Maki M., Leung K.T., Qin W. (2009). The prospects of cellulase-producing bacteria for the bioconversion of lignocellulosic biomass. Int. J. Biol. Sci..

[185] Naik S.N., Goud V., Rout P.K., Dalai A.K. (2010). Production of first and second generation biofuels: A comprehensive review. Renew. Sust. Energ. Rev..

[186] Kasana R.C. (2010). Proteases from psychrotrophs: An overview. Crit. Rev. Microbiol..

[187] Kumar V., Satyanarayana T., Seckbach J., Oren A., Stan-Latter H. (2013). Thermoalkaliphilic microbes. Polyextremophiles: Life under Multiple Forms of Stress.

[188] Duffaud G.D., McCutchen C.M., Leduc P., Parker K.N., Kelly R.M. (1997). Purification and characterization of extremely thermostable beta-mannanase, beta-mannosidase, and alpha-galactosidase from the hyperthermophilic eubacterium *Thermotoga neapolitana* 5068. Appl. Environ. Microbiol..

[189] Matsui I., Sakai Y., Matsui E., Kikuchi H., Kawarabayasi Y., Honda K. (2000). Novel substrate specificity of a membrane-bound beta-glycosidase from the hyperthermophilic archaeon *Pyrococcus horikoshii*. FEBS Lett..

[190] Goyal K., Selvakumar P., Hayashi K. (2001). Characterization of a thermostable beta-glucosidase (Bg1B) from *Thermotoga maritima* showing transglycosylation activity. J. Mol. Catal. B-Enzym..

[191] Subramaniyan S., Prema P. (2002). Biotechnology of microbial xylanases: Enzymology, molecular biology, and application. Crit. Rev. Biotechnol..

[192] Ooteghem V.S. (2005). Process for Generation of Hydrogen Gas from Various Feedstocks Using Thermophilic Bacteria.

[193] Jiang Z.Q., Deng W., Zhu Y.P., Li L.T., Sheng Y.J., Hayashi K. (2004). The recombinant xylanase B of T*hermotoga maritima* is highly xylan specific and produces exclusively xylobiose from xylans, a unique character for industrial applications. J. Mol. Catal. B-Enzym..

[194] Kang H.J., Uegaki K., Fukada H., Ishikawa K. (2007). Improvement of the enzymatic activity of the hyperthermophilic cellulase from *Pyrococcus horikoshii*. Extremophiles.

[195] Mao X., Hong Y., Shao Z., Zhao Y., Liu Z. (2010). A novel cold-active and alkali-stable beta-glucosidase gene isolated from the marine bacterium *Martelella mediterranea*. Appl. Biochem. Biotechnol..

[196] Chen S., Hong Y., Shao Z., Liu Z. (2010). A cold-active β-glucosidase (Bgl1C) from a sea bacteria *Exiguobacterium oxidotolerans* A011. World J. Microb. Biot..

[197] Garsoux G., Lamotte J., Gerday C., Feller G. (2004). Kinetic and structural optimization to catalysis at low temperatures in a psychrophilic cellulase from the Antarctic bacterium *Pseudoalteromonas haloplanktis*. Biochem. J..

[198] Collins T., Hoyoux A., Dutron A., Georis J., Genot B., Dauvrin T., Arnaut F., Gerday C., Feller G. (2006). Use of glycoside hydrolase family 8 xylanases in baking. J. Cereal Sci..

[199] Dornez E., Verjans P., Arnaut F., Delcour J.A., Courtin C.M. (2011). Use of psychrophilic xylanases provides insight into the xylanase functionality in bread making. J. Agric. Food Chem..

[200] Hildebrandt P., Wanarska M., Kur J. (2009). A new cold-adapted beta-D-galactosidase from the Antarctic *Arthrobacter* sp. 32c—Gene cloning, overexpression, purification and properties. BMC Microbiol..

[201] Varbanets L.D., Avdeeva L.V., Borzova N.V., Matseliukh E.V., Gudzenko A.V., Kiprianova E.A., Iaroshenko L.V. (2011). The Black Sea bacteria—Producers of hydrolytic enzymes. Mikrobiol. Z.

[202] Rawlings N.D., Waller M., Barrett A.J., Bateman A. (2014). MEROPS: The database of proteolytic enzymes, their substrates and inhibitors. Nucleic Acids. Res..

[203] Lopez-Otin C., Matrisian L.M. (2007). Emerging roles of proteases in tumour suppression. Nat. Rev. Cancer.

[204] Rawlings N.D., Bateman A. (2009). Pepsin homologues in bacteria. BMC Genomics.

[205] Gunbin K.V., Afonnikov D.A., Kolchanov N.A. (2009). Molecular evolution of the hyperthermophilic archaea of the *Pyrococcus* genus: Analysis of adaptation to different environmental conditions. BMC Genomics.

[206] Stetter K.O. (2006). History of discovery of the first hyperthermophiles. Extremophiles.

[207] Halio S.B., Bauer M.W., Mukund S., Adams M., Kelly R.M. (1997). Purification and characterization of two functional forms of intracellular protease PfpI from the hyperthermophilic archaeon *Pyrococcus furiosus*. Appl. Environ. Microbiol..

[208] Ward D.E., Shockley K.R., Chang L.S., Levy R.D., Michel J.K., Conners S.B., Kelly R.M. (2002). Proteolysis in hyperthermophilic microorganisms. Archaea.

[209] Atomi H. (2005). Recent progress towards the application of hyperthermophiles and their enzymes. Curr. Opin. Chem. Biol..

[210] Cavicchioli R., Siddiqui K.S., Andrews D., Sowers K.R. (2002). Low-temperature extremophiles and their applications. Curr. Opin. Biotechnol..

[211] Gerday C., Aittaleb M., Bentahir M., Chessa J.P., Claverie P., Collins T., D’Amico S., Dumont J., Garsoux G., Georlette D. (2000). Cold-adapted enzymes: From fundamentals to biotechnology. Trends Biotechnol..

[212] Cristobal H.A., Lopez M.A., Kothe E., Abate C.M. (2011). Diversity of protease-producing marine bacteria from sub-antarctic environments. J. Basic Microbiol..

[213] Kulakova L., Galkin A., Kurihara T., Yoshimura T., Esaki N. (1999). Cold-active serine alkaline protease from the psychrotrophic bacterium *Shewanella* strain ac10: Gene cloning and enzyme purification and characterization. Appl. Environ. Microbiol..

[214] Huston A.L., Methe B., Deming J.W. (2004). Purification, characterization, and sequencing of an extracellular cold-active aminopeptidase produced by marine psychrophile *Colwellia psychrerythraea* strain 34H. Appl. Environ. Microbiol..

[215] Sastre D.E., Paggi R.A., de Castro R.E. (2011). The Lon protease from the haloalkaliphilic archaeon *Natrialba magadii* is transcriptionally linked to a cluster of putative membrane proteases and displays DNA-binding activity. Microbiol. Res..

[216] Cerletti M., Martinez M.J., Gimenez M.I., Sastre D.E., Paggi R.A., de Castro R.E. (2014). The LonB protease controls membrane lipids composition and is essential for viability in the extremophilic haloarchaeon *Haloferax volcanii*. Environ. Microbiol..

[217] Gimenez M.I., Studdert C.A., Sanchez J.J., De Castro R.E. (2000). Extracellular protease of *Natrialba magadii*: Purification and biochemical characterization. Extremophiles.

[218] Kamekura M., Seno Y., Dyall-Smith M. (1996). Halolysin R4, a serine proteinase from the halophilic archaeon *Haloferax mediterranei*; gene cloning, expression and structural studies. Biochim. Biophys. Acta.

[219] De Castro R.E., Maupin-Furlow J.A., Gimenez M.I., Herrera Seitz M.K., Sanchez J.J. (2006). Haloarchaeal proteases and proteolytic systems. FEMS Microbiol. Rev..

[220] Vidyasagar M., Prakash S., Mahajan V., Shouche Y.S., Sreeramulu K. (2009). Purification and characterization of an extreme halothermophilic protease from a halophilic bacterium *Chromohalobacter* sp. TVSP101. Braz. J. Microbiol..

[221] Hough D.W., Danson M.J. (1999). Extremozymes. Curr. Opin. Chem. Biol..

[222] Asker D., Awad T.S., Beppu T., Ueda K. (2008). *Deinococcus misasensis* and *Deinococcus roseus*, novel members of the genus *Deinococcus*, isolated from a radioactive site in Japan. Syst. Appl. Microbiol..

[223] Asker D., Awad T.S., McLandsborough L., Beppu T., Ueda K. (2011). *Deinococcus depolymerans* sp. nov., a gamma- and UV-radiation-resistant bacterium, isolated from a naturally radioactive site. Int. J. Syst. Evol. Microbiol..

[224] Kampfer P., Lodders N., Huber B., Falsen E., Busse H.J. (2008). *Deinococcus aquatilis* sp. nov., isolated from water. Int. J. Syst. Evol. Microbiol..

[225] Asker D., Awad T.S., Beppu T., Ueda K. (2009). *Deinococcus aquiradiocola* sp. nov., isolated from a radioactive site in Japan. Int. J. Syst. Evol. Microbiol..

[226] Suresh K., Reddy G.S., Sengupta S., Shivaji S. (2004). *Deinococcus indicus* sp. nov., an arsenic-resistant bacterium from an aquifer in West Bengal, India. Int. J. Syst. Evol. Microbiol..

[227] Kimura H., Asada R., Masta A., Naganuma T. (2003). Distribution of microorganisms in the subsurface of the manus basin hydrothermal vent field in Papua New Guinea. Appl. Environ. Microbiol..

[228] Liedert C., Peltola M., Bernhardt J., Neubauer P., Salkinoja-Salonen M. (2012). Physiology of resistant *Deinococcus geothermalis* bacterium aerobically cultivated in low-manganese medium. J. Bacteriol..

[229] Pietrow O., Panek A., Synowiecki J. (2013). Extracellular proteolytic activity of *Deinococcus geothermalis*. Afr. J. Biotechnol..

[230] Friedrich A.B., Antranikian G. (1996). Keratin degradation by *Fervidobacterium pennavorans*, a novel thermophilic anaerobic species of the order thermotogales. Appl. Environ. Microbiol..

[231] Burlini N., Magnani P., Villa A., Macchi F., Tortora P., Guerritore A. (1992). A heat-stable serine proteinase from the extreme thermophilic archaebacterium *Sulfolobus solfataricus*. Biochim. Biophys. Acta.

[232] Klingeberg M., Hashwa F., Antranikian G. (1991). Properties of extremely thermostable proteases from anaerobic hyperthermophilic bacteria. Appl. Microbiol. Biotechnol..

[233] Jang H.J., Kim B.C., Pyun Y.R., Kim Y.S. (2002). A novel subtilisin-like serine protease from *Thermoanaerobacter yonseiensis* KB-1: Its cloning, expression, and biochemical properties. Extremophiles.

[234] Lloyd K.G., Schreiber L., Petersen D.G., Kjeldsen K.U., Lever M.A., Steen A.D., Stepanauskas R., Richter M., Kleindienst S., Lenk S. (2013). Predominant archaea in marine sediments degrade detrital proteins. Nature.

[235] Seth-Pasricha M., Bidle K.A., Bidle K.D. (2013). Specificity of archaeal caspase activity in the extreme halophile *Haloferax volcanii*. Environ. Microbiol. Rep..

[236] Arpigny J.L., Jaeger K.E. (1999). Bacterial lipolytic enzymes: Classification and properties. Biochem. J..

[237] de Pascale D., Cusano A.M., Autore F., Parrilli E., di Prisco G., Marino G., Tutino M.L. (2008). The cold-active Lip1 lipase from the Antarctic bacterium *Pseudoalteromonas haloplanktis* TAC125 is a member of a new bacterial lipolytic enzyme family. Extremophiles.

[238] Gupta R., Gupta N., Rathi P. (2004). Bacterial lipases: An overview of production, purification and biochemical properties. Appl. Microbiol. Biotechnol..

[239] Hasan F., Shah A.A., Hameed A. (2006). Industrial applications of microbial lipases. Enzyme Microb. Technol..

[240] Jaeger K.E., Eggert T. (2002). Lipases for biotechnology. Curr. Opin. Biotechnol..

[241] Sangeetha R., Arulpandi I., Geetha A. (2011). Bacterial lipases as potential industrial biocatalysts: An overview. Res. J. Microbiol..

[242] Charpe T.W., Rathod V.K. (2011). Biodiesel production using waste frying oil. Waste Manag..

[243] Gupta P., Upadhyay L.S.B., Shrivastava R. (2011). Lipase catalyzed-transesterification of vegetable oils by lipolytic bacteria. Res. J. Microbiol..

[244] Bajaj A., Lohan P., Jha P.N., Mehrotra R. (2010). Biodiesel production through lipase catalyzed transesterification: An overview. J. Mol. Catal. B: Enzym..

[245] Reetz M.T., Jaeger K.E. (1998). Overexpression, immobilization and biotechnological application of *Pseudomonas* lipases. Chem. Phys. Lipids.

[246] Fucinos P., Gonzalez R., Atanes E., Sestelo A.B., Perez-Guerra N., Pastrana L., Rua M.L. (2012). Lipases and esterases from extremophiles: Overview and case example of the production and purification of an esterase from *Thermus thermophilus* HB27. Methods Mol. Biol..

[247] Andualema B., Gessesse A. (2012). Microbial lipases and their industrial applications: Review. Biotechnol. Appl. Biochem..

[248] Schafer T., Antranikian G., Royter M., Hoff T. (2011). Lipases from Thermophilic Anaerobes.

[249] Vind J., Knötzel J.C.F., Borch K., Svendsen A., Callisen T.H., Yaver D., Bjornvad M.E., Hansen P.K., Lamsa M. (2012). Lipase Variants. US.

[250] Mardanov A.V., Ravin N.V., Svetlitchnyi V.A., Beletsky A.V., Miroshnichenko M.L., Bonch-Osmolovskaya E.A., Skryabin K.G. (2009). Metabolic versatility and indigenous origin of the archaeon *Thermococcus sibiricus*, isolated from a siberian oil reservoir, as revealed by genome analysis. Appl. Environ. Microbiol..

[251] Kim S.B., Lee W., Ryu Y.W. (2008). Cloning and characterization of thermostable esterase from *Archaeoglobus fulgidus*. J. Microbiol..

[252] Hotta Y., Ezaki S., Atomi H., Imanaka T. (2002). Extremely stable and versatile carboxylesterase from a hyperthermophilic archaeon. Appl. Environ. Microbiol..

[253] Park Y.J., Yoon S.J., Lee H.B. (2008). A novel thermostable arylesterase from the archaeon *Sulfolobus solfataricus* P1: Purification, characterization, and expression. J. Bacteriol..

[254] Arpigny J.L., Jendrossek D., Jaeger K.E. (1998). A novel heat-stable lipolytic enzyme from *Sulfolobus acidocaldarius* DSM 639 displaying similarity to polyhydroxyalkanoate depolymerases. FEMS Microbiol. Lett..

[255] Huddleston S., Yallop C.A., Charalambous B.M. (1995). The identification and partial characterisation of a novel inducible extracellular thermostable esterase from the archaeon *Sulfolobus shibatae*. Biochem. Biophys. Res. Commun..

[256] Ikeda M., Clark D.S. (1998). Molecular cloning of extremely thermostable esterase gene from hyperthermophilic archaeon *Pyrococcus furiosus* in *Escherichia coli*. Biotechnol. Bioeng..

[257] Ryu H.S., Kim H.K., Choi W.C., Kim M.H., Park S.Y., Han N.S., Oh T.K., Lee J.K. (2006). New cold-adapted lipase from *Photobacterium lipolyticum* sp. nov. that is closely related to filamentous fungal lipases. Appl. Microbiol. Biotechnol..

[258] Bosley J.A., Peilow A.D. (1993). Preparation of Immobilized Lipase by Adsorption of Lipase and a Non-Lipase Protein on a Support.

[259] Yang K.S., Sohn J.H., Kim H.K. (2009). Catalytic properties of a lipase from *Photobacterium lipolyticum* for biodiesel production containing a high methanol concentration. J. Biosci. Bioeng..

[260] Gotor-Fernández V., Busto E., Gotor V. (2006). *Candida antarctica* Lipase B: An ideal biocatalyst for the preparation of nitrogenated organic compounds. Adv. Synth. Catal..

[261] Qin B., Liang P., Jia X., Zhang X., Mu M., Wang X.-Y., Ma G.-Z., Jin D.-N., You S. (2013). Directed evolution of *Candida antarctica* lipase B for kinetic resolution of profen esters. Catal. Commun..

[262] Singh N., Jha M.K., Sarma A.K., Kumar S., Sarma A.K., Tyagi S.K., Yadav Y.K. (2014). A critical review of enzymatic transesterification: A sustainable technology for biodiesel production. Recent Advances in Bioenergy Research.

[263] Nielsen T.B., Ishii M., Kirk O., Margesin R., Schinner F. (1999). Lipases A and B from the yeast *Candida antarctica*. Biotechnological Applications of Cold-Adapted Organisms.

[264] Widersten M. (2014). Protein engineering for development of new hydrolytic biocatalysts. Curr. Opin. Chem. Biol..

[265] Lo Giudice A., Michaud L., de Pascale D., de Domenico M., di Prisco G., Fani R., Bruni V. (2006). Lipolytic activity of Antarctic cold-adapted marine bacteria (Terra Nova Bay, Ross Sea). J. Appl. Microbiol..

[266] Al Khudary R., Venkatachalam R., Katzer M., Elleuche S., Antranikian G. (2010). A cold-adapted esterase of a novel marine isolate, *Pseudoalteromonas arctica*: Gene cloning, enzyme purification and characterization. Extremophiles.

[267] Rabus R., Ruepp A., Frickey T., Rattei T., Fartmann B., Stark M., Bauer M., Zibat A., Lombardot T., Becker I. (2004). The genome of *Desulfotalea psychrophila*, a sulfate-reducing bacterium from permanently cold Arctic sediments. Environ. Microbiol..

[268] Zeng X., Xiao X., Wang P., Wang F. (2004). Screening and characterization of psychrotrophic lipolytic bacteria from deep-sea sediments. J. Microbiol. Biotechnol..

[269] Do H., Lee J.H., Kwon M.H., Song H.E., An J.Y., Eom S.H., Lee S.G., Kim H.J. (2013). Purification, characterization and preliminary X-ray diffraction analysis of a cold-active lipase (CpsLip) from the psychrophilic bacterium *Colwellia psychrerythraea* 34H. Acta Crystallogr. Sect. F. Struct. Biol. Cryst. Commun..

[270] Amoozegar M.A., Salehghamari E., Khajeh K., Kabiri M., Naddaf S. (2008). Production of an extracellular thermohalophilic lipase from a moderately halophilic bacterium, *Salinivibrio* sp. strain SA-2. J. Basic Microbiol..

[271] Córdova-López J., Camacho-Córdova D.I., Camacho-Ruiz R.M., Mateos J.C., Rodríguez J. (2014). *Haloarcula marismortui*, eighty-four years after its discovery in the Dead Sea, Review. IJERT.

[272] Muller-Santos M., de Souza E.M., Pedrosa Fde O., Mitchell D.A., Longhi S., Carriere F., Canaan S., Krieger N. (2009). First evidence for the salt-dependent folding and activity of an esterase from the halophilic archaea *Haloarcula marismortui*. Biochim. Biophys. Acta.

[273] Corral P., Gutierrez M.C., Castillo A.M., Dominguez M., Lopalco P., Corcelli A., Ventosa A. (2013). *Natronococcus roseus* sp. nov., a haloalkaliphilic archaeon from a hypersaline lake. Int. J. Syst. Evol. Microbiol..

